# Dynamic eIF3‐S6 Phase Separation Switch Instructed by m^6^A Modification Drives the Molting of Locusts

**DOI:** 10.1002/advs.202510505

**Published:** 2025-07-28

**Authors:** Zhihao Hu, Yu Zhou, Huimin Wang, Yaxi Wu, Rui Han, Shili Liu, Feng Jiang, Xiaojiao Guo, Meiling Yang

**Affiliations:** ^1^ College of Life Sciences Capital Normal University Beijing 100048 China; ^2^ State Key Laboratory of Animal Biodiversity Conservation and Integrated Pest Management Institute of Zoology Chinese Academy of Sciences Beijing 100101 China

**Keywords:** liquid–liquid phase separation, locusts, m^6^A modification, molting

## Abstract

Molting is critical for the development and growth of insects. A central juncture in molting is the periodic and coordinated regulation of molting‐responsive mRNAs, which are essential for degrading the old cuticle and forming a new one. Knowledge is lacking of how molting‐responsive molecules function cooperatively and timely. Here, multi‐stage m^6^A modification levels, m^6^A reader condensation, and transcriptome analyses are developed to track the dynamic functional coupling for molting signals. The results showed that the molting‐responsive mRNAs are downregulated with the decrease of m^6^A modification levels from pre‐molt to the molt stage. The eIF3‐S6 is further identified, as an m^6^A reader, can directly bind to *EcR* A^335^ and *Cht10* A^7589^. The low mRNA m^6^A modification in the molt stage facilitates eIF3‐S6 to undergo liquid–liquid phase separation (LLPS) by binding m^6^A‐containing *EcR*/*Cht10* into condensed droplets for stabilizing the mRNAs. Disruption of m^6^A modification or breaking phase separation can cause a disorder of the new/old cuticle synthesis/degradation in molting. The disorder results in abnormal and unsuccessful molting, ultimately leading to the death of nymphs. Overall, the data mechanistically highlight an example of how two central machineries, m^6^A modification and LLPS, can accurately and functionally cooperate to optimize molting‐responsive gene expression for insect molting.

## Graphical abstract

This research reveals that reduced m^6^A modifications during insect molting trigger liquid–liquid phase separation (LLPS) of the m^6^A reader eIF3‐S6, which binds and stabilizes key molting mRNAs, including *EcR* and *Cht10*. Disrupting m^6^A or LLPS causes cuticle remodeling defects and molting failure, highlighting the synergistic control of gene expression by m^6^A methylation and phase separation.

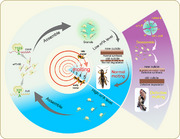

## 1 Introduction

The molting is critical for the development and growth of insects. The removal of superfluous and obsolete old cuticle and the remodeling of new cuticle are pivotal processes,^[^ ^1^, ^2^ ^]^ in which periodic and coordinated regulation of molting‐responsive mRNAs is essential for molting development.^[^ ^3^ ^]^ Indeed, a significant proportion of molting‐responsive mRNAs was highest during intermolt and premolt stages and lowest at postmolt stage,^[^ ^4^ ^]^ but several mRNAs are not necessary for molting development and even be harmful to molting at the late stage.^[^ ^5^ ^]^ Therefore, a central but understudied question in molting is how molting‐responsive molecules function cooperatively and timely within a narrow time window during molting development.

The migratory locust *Locusta migratoria*, a worldwide pest species, undergoes five molting stages in its life cycle and has been widely used as a model for studying molting development.^[^ ^6^ ^]^ Ecdysone signaling functions as a long‐range critical signal to generate temporal‐specific molting responses and coordinate the degeneration or remodeling processes during the locust molting.^[^ ^7^, ^8^ ^]^ Specifically, the active form of ecdysone, 20‐hydroxyecdysone (20E) binds to the heterodimeric nuclear hormone receptors, ecdysone receptor (EcR),^[^ ^9^ ^]^ to transmit signals down, directly induces transcription of early 20E‐response genes such as BR‐C, E74A, and E75A.^[^ ^7^ ^]^ Subsequently, these early genes further transduce and amplify the ecdysone signal to induce cell death by regulating a group of secondary‐response genes associated with both apoptosis and remodeling.^[^ ^10^ ^]^ Indeed, several studies demonstrate that many secondary‐response genes, such as *chitin synthase1*, *chitinase10, sinuous, Tweedle, Abd‐9, UDP‐N‐acetylglucosamine pyrophosphorylases*, *Chitin Deacetylase2*, control the molting process, and knockdown of these molting‐responsive genes leads to a significant reduction in survival rate along with a molting defect of the locust.^[^ ^5^, ^11^, ^12^, ^13^, ^14^ ^]^ However, these studies do not provide information on direct spatiotemporal regulation of transcriptional responses to drive the timely and precise expression of molting‐responsive genes and to ensure a delicate balance between the degradation and remodeling of the cuticle during insect molting.

RNA epigenetic modifications can reversibly fine‐tune transcriptional plasticity, providing the flexibility needed to orchestrate gene expression.^[^ ^15^ ^]^ Among numerous RNA modifications, *N* ^6^‐methyladenosine (m^6^A), 5‐methylcytidine (m^5^C), *N* ^7^‐methylguanosine (m^7^G), and *N* ^1^‐methyladenosine (m^1^A) have been associated with cell proliferation, differentiation, invasion, migration, stemness, and metabolism.^[^ ^16^, ^17^, ^18^, ^19^, ^20^, ^21^ ^]^ *N* ^6^‐methyladenosine (m^6^A) is the most abundant type of modification in eukaryotic RNAs and plays important roles in multiple fundamental biological processes.^[^ ^22^, ^23^, ^24^, ^25^ ^]^ This modification is a reversible process regulated by “writers” (methyltransferases) and “erasers” (demethylases), responsible for positioning and removing the covalent chemical modifications, respectively,^[^ ^26^, ^27^ ^]^ and “readers”, downstream regulators that recognize and bind specific modifications.^[^ ^28^ ^]^ Emerging evidence suggests that m^6^A methylation orchestrates the expression of a large number of developmentally regulated genes through controlling the balance by promoting or repressing the expression of the targeted locus in diverse programmed cell death processes.^[^ ^29^, ^30^ ^]^ Given that RNA‐mediated epigenetic regulation is a fundamental evolution strategy for orchestrating gene expression in a timely and reversible manner. We suspect whether this regulation impacts cuticle tissue removal/remodeling and how its alteration contributes to the balance between molting‐responsive transcripts degradation and stability in the normal molting development.

In this study, we explored the dynamic interplay between the transcription of molting‐responsive RNAs and m^6^A modification across the genome during the locust molting process. We simultaneously monitored both the transcription levels and m^6^A modification levels to understand their functional coupling. Our findings revealed that molting‐responsive RNAs were downregulated in concert with a decrease in mRNA m^6^A modification levels as locusts develop from the pre‐molt to the molt stage. We identified a crucial m^6^A reader in the integument that can bind to two molting‐responsive m^6^A‐modified mRNAs. Furthermore, we observed that m^6^A reader condensation coupling dynamically occurred during molting, facilitated by the incorporation of low m^6^A‐modified RNAs into granules. Collectively, these regulatory mechanisms enhance mRNA stability, and shed light on how central regulation machineries communicate and coordinate to optimize the expression of molting‐responsive genes for insect molting.

## 2 Results

### 2.1 The m^6^A Modification Dynamically Responds to Locusts Molting Process

**Figure 1:**
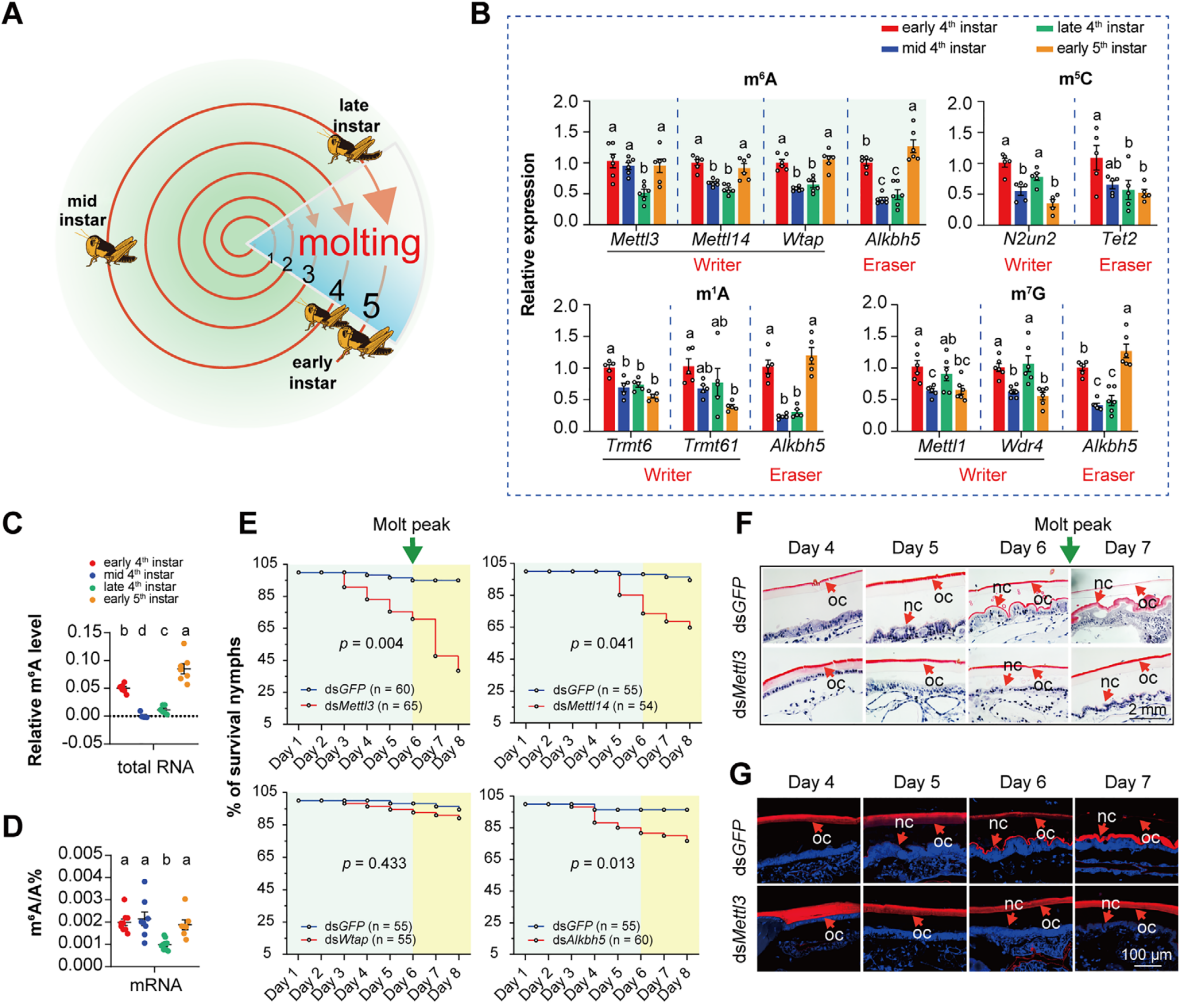
The m^6^A modification dynamically responds to the locusts molting process. A) The locust undergoes five molting stages in its life cycle. Each nymph progresses through early‐stage, mid‐stage, late‐stage, and ends with molting to the early‐stage of the next instar nymph, thereby completing one instar cycle. B) Relative expression levels of the “writer” and “eraser” genes associated with m^1^A, m^7^G, m^5^C, and m^6^A RNA modifications. C, D) Total RNA m^6^A modification levels (C, n = 7) detected using colorimetry assays and the mRNA m^6^A modification levels (D, n = 8) detected using HPLC‐MS assays in the integuments of early‐stage, mid‐stage, and late‐stage of the 4^th^ instar nymph and early‐stage of the 5^th^ instar nymph. E) Effects of silencing *Mettl3*, *Mettl14*, *Wtap*, and *Alkbh5* on locust mortality. The green arrow indicates the time for molting. F,G) Effects of silencing *Mettl3* on the metabolism of new and old cuticle using Hematoxylin and eosin staining (F) and chitin staining (G). OC, old cuticle, NC, new cuticle. Different letters indicate statistically significant differences between groups using one‐way ANOVA (Tukey's multiple comparisons test, *p* < 0.05) (B–D). *P* values in survival curve were determined by the Kaplan–Meier statistical analysis (E). Data are presented as mean ± SEM.

Locusts undergo five molting stages from egg to adult, with each nymphal instar concluding with the molting process, which involves shedding and replacing their rigid exoskeleton (**Figure** **1**A). To explore whether epigenetic modifications of RNA are associated with the molting process in locusts, we detected several prevalent modifications in organisms: m^1^A, m^7^G, m^5^C, and m^6^A. Employing qPCR assays, we first assessed the expression patterns of the enzymes responsible for adding (“writers”) and removing (“erasers”) these modifications during molting. The expression levels of m^6^A “writers” (*Mettl3*, *Mettl14* and *Wtap*), and the putative “eraser” (*Alkbh5*), exhibited a similar wave‐like pattern, peaking at the early stage of the 4^th^ or 5^th^ instar (pre‐molt stage), and then dropping to their lowest level at the late stage of instar (molt stage). Conversely, the mRNA expression levels of m^5^C “writer” (*N2un2*), m^1^A “writer” (*Trmt6* and *Trmt61*) and m^7^G “writer” (*Mettl1* and *Wdr4*) exhibited a down‐regulated pattern from the early 4^th^ instar to the early 5^th^ instar nymph stage, which did not correlate with the molting process (Figure 1B), although the “eraser” *Alkbh5*, which was suggested as the potential demethylase by the homology alignment and structural analyses (Figure S1, Supporting Information), can remove m^6^A, m^1^A and m^7^G modifications,^[^ ^31^, ^32^ ^]^ showed a similar trend. Thus, we speculated that m^6^A modification is specifically associated with molting in locusts.

We further employed a colorimetric ELISA‐like assay and high‐performance liquid chromatography tandem mass spectrometry (HPLC‐MS/MS) to measure the levels of m^6^A modifications in the integument of nymphs at various stages of each molting cycle, especially during the early, mid, and late stages of the 4^th^ instar nymph, as well as the early‐stage of the 5^th^ instar nymph. Both colorimetric and HPLC‐MS/MS assays indicated that the levels of m^6^A modifications in total RNA and mRNA levels mirrored the expression patterns of m^6^A writers and the eraser, peaking at the early stage, and progressively diminishing to their lowest levels by the late‐stage (Figure 1C,D; Figure S2A, Supporting Information). Thus, the RNA m^6^A modification exhibits dynamic fluctuations throughout the molting process in locusts.

To determine the role of m^6^A modification in the molting process, we conducted RNA interference (RNAi) experiments targeting *Mettl3*, *Mettl14*, *Wtap*, and *Alkbh5*, respectively, by microinjecting dsRNA three times at two‐day intervals into 4^th^ instar nymphs. The transcript levels of these four genes were significantly reduced at 48 h post‐injection (Figure S2B, Supporting Information). Locusts injected with ds*Mettl3* displayed a distinct lethal phenotype caused by unsuccessful molting. Out of 65 fourth‐instar nymphs injected with ds*Mettl3*, 48 (73.8%) failed to develop into the 5^th^ instar nymphs and ultimately died during the molting stage, compared to only 5% mortality in the control group (Figure 1E). By contrast, after injection of ds*Mettl14*, ds*Wtap*, and ds*Alkbh5*, the mortality rates were only 35.2%, 12.7% and 31.7%, respectively, compared to the control rates of 5.5%, 5.5%, and 3.6% during the molting process (Figure 1E).

Given that the pronounced effects of ds*Mettl3* on molting compared to ds*Mettl14*, ds*Wtap*, and ds*Alkbh5*, subsequent experiments concentrated on *Mettl3* to investigate the role of m^6^A modification in locust molting. To determine the cause of molting‐related mortality following *Mettl3* RNAi knockdown, we first measured m^6^A level in the integuments of locusts using dot blot assays with m^6^A specific antibody. The results indicated that knockdown of *Mettl3* decreased the m^6^A levels in total RNA by ≈83% (*t* = 3.482, *p* < 0.05, Figure S2C, Supporting Information). In contrast, knockdown of *Mettl14* did not significantly change the m^6^A level (*t* = 0.2499, *p* > 0.05, Figure S2D, Supporting Information). We then determined the effects on cuticle formation following *Mettl3* knockdown. Histological sections of the integuments of ds*GFP*‐ and ds*Mettl3*‐injected nymphs from day 4 to day 7 were prepared for H&E and chitin staining. Both H&E and chitin staining indicated that the progresses of apolysis, new cuticle synthesis, and old cuticle degradation were delayed or impaired in nymphs injected with ds*Mettl3*, compared to ds*GFP* control. Notably, at day 4 of 4^th^ instar nymphs (N4D4), apolysis was delayed in ds*Mettl3* treated nymphs. By day 5 (N4D5), new cuticle formation was initiated in the control group, but was absent in ds*Mettl3‐*injected locusts. By day 6(N4D6), the new multi‐lamellar cuticle was synthesized normally in the control group, yet was still absent in ds*Mettl3*‐injected locusts. Finally, by day 7 (N4D7), the old cuticle was significantly degraded in control insects, whereas ds*Mettl3*‐injected insects retained a thick, lamellar old cuticle structure. Altogether, nymphs injected with ds*Mettl3* failed to molt on time and remained in the nymph stage until death. These findings imply a critical role for m^6^A modification in the molting process (Figure 1F,G).

### 2.2 *EcR* and *Cht10* m^6^A Modifications are Dynamic and Associated with Molting

**Figure 2:**
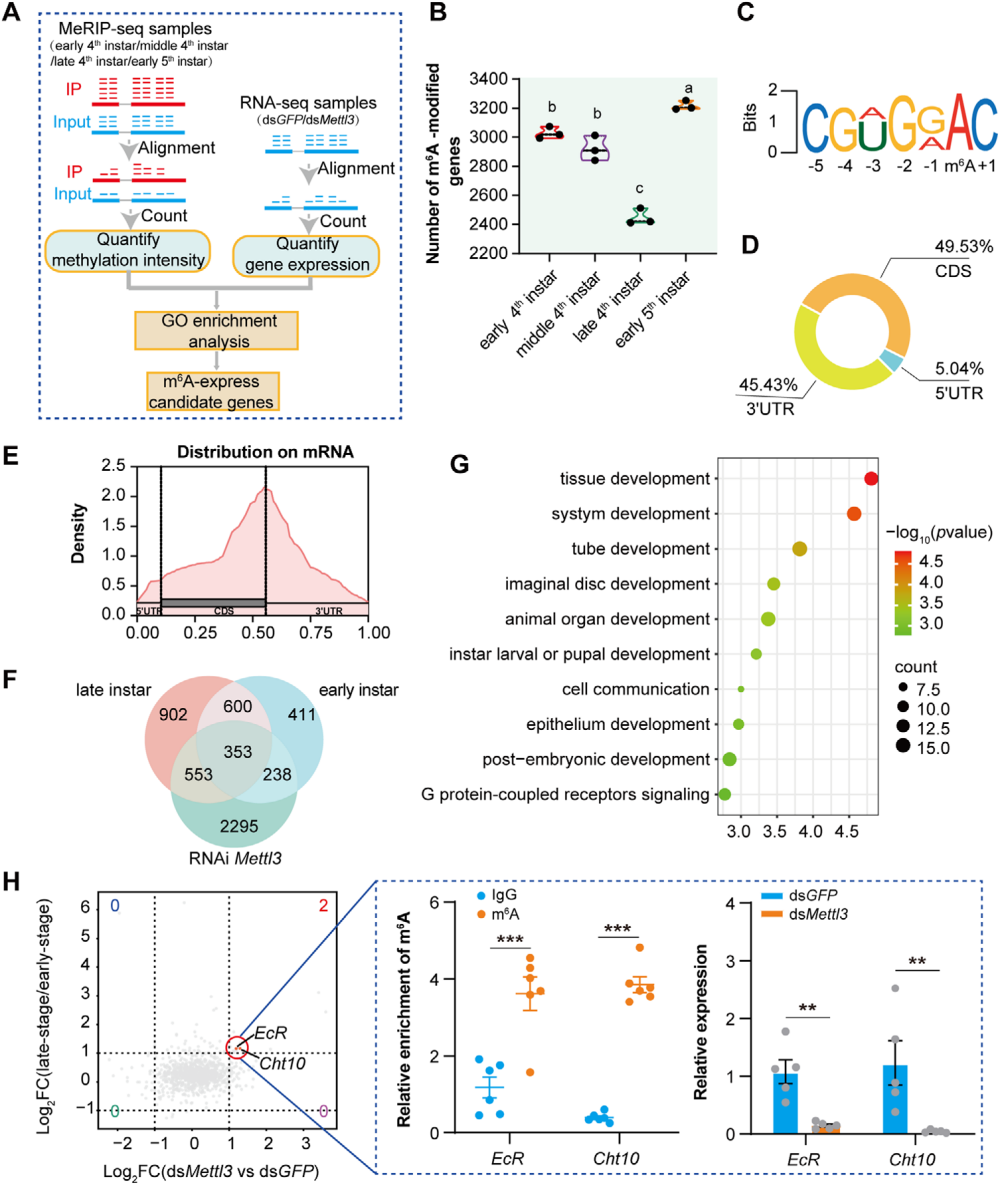
*EcR* and *Cht10* m^6^A modifications are dynamic and associated with molting. A) Schematic of MeRIP‐seq and RNA‐seq analysis. B) The number of m^6^A‐modified genes identified in the integuments of early‐stage, mid‐stage, and late‐stage of the 4^th^ instar nymph and early‐stage of the 5^th^ instar nymph by MeRIP‐seq analysis. C) Representative sequence motif identified from the m^6^A peaks in the m^6^A IP group. D) Pie chart showing the distribution of m^6^A peaks across gene regions (CDS, 5′UTR, and 3′UTR) in the locust integuments from early‐stage, mid‐stage, and late‐stage of the 4^th^ instar nymph and early‐stage of the 5^th^ instar nymph. E) Metagene profiles depicting the distribution of m^6^A across the transcriptome of the locust integuments from early‐stage, mid‐stage, and late‐stage of the 4^th^ instar nymph and early‐stage of the 5^th^ instar nymph. F) The number of common and specific m^6^A‐containing genes identified through the combined analysis of two paired samples from MeRIP‐seq (early‐stage vs late‐stage) and RNA‐seq (ds*Mettl3* and ds*GFP*). G) Biological pathways enriched by Gene Ontology analysis for the identified common set of 353 m^6^A‐containing differentially expressed genes from the combined analysis of MeRIP‐seq and RNA‐seq. H) Quadrant diagram displaying genes with significant reductions in both m^6^A enrichment (late vs early stage, MeRIP‐seq) and transcript levels (ds*Mettl3*‐ vs ds*GFP*‐injected nymphs, RNA‐seq). Red highlighted boxes indicate the top candidate targets, *EcR* and *Cht10*, showing > 3‐fold downregulation upon *Mettl3* knockdown. The m^6^A levels and expression levels of *EcR* and *Cht10* were verified by m^6^A‐IP‐qPCR (n = 6) and qPCR (n = 5) analysis in the inset dashed panel. *P* values were determined by a two‐tailed unpaired *t*‐test. Data are presented as mean ± SEM. ^**^ *p* < 0.01, ^***^ *p* < 0.001.

To further explore the relationship between m^6^A RNA modification and molting, we first generated a comprehensive m^6^A profile across the transcriptome by employing methylated RNA immunoprecipitation sequencing (MeRIP‐seq) in the integument of nymphs at different developmental stages: early, mid, late 4^th^ instar, and early 5^th^ instar. We identified a comparable number of transcripts marked by m^6^A peaks in the early and mid 4^th^ instar and early 5^th^ instar nymphs, contrasting with a notably lower count of m^6^A transcripts in the late 4^th^ instar nymphs (one‐way ANOVA, *F* = 96.44, *p* <0.0001; **Figure** **2**B; Figure S3A, Supporting Information). We observed a significant enrichment of m^6^A peaks for the conserved RRACH motif (R is A/G and H is A/C/U) within the MeRIP group (Figure 2C; Figure S3D, Supporting Information). When examining the distribution of m^6^A across different stages, we found a preference for m^6^A peaks to occur within coding sequence (CDS) regions and 3′ untranslated region (3′ UTR), accounting for ≈50% and 45% of the confidently detected m^6^A peaks, respectively (Figure 2D). Given that CDS and UTR segments vary in length, we conducted an unbiased enrichment analysis by normalizing to the typical sizes of each segment. The stop codon region emerged as the segment most enriched with m^6^A across all samples (Figure 2E).

To investigate the specific m^6^A‐containing genes related to locust molting, we performed RNA‐seq on integuments of nymphs injected with ds*Mettl3* and ds*GFP*, respectively, and analyzed the differentially expressed genes (DEGs) (Figure 2A). We then compared m^6^A‐containing genes in two paired samples from MeRIP‐seq data (early‐stage vs late‐stage), observing an increasing m^6^A modification trend in transcripts that preferentially exhibited significantly differential expression between RNA‐seq samples (ds*Mettl3* and ds*GFP*) (Figure S3A–C, Supporting Information). Among these transcripts, 353 DEGs were found to be common between the two transcriptomes (Figure 2F).

To determine which biological pathways were enriched for the identified common 353 m^6^A‐bearing DEGs, we performed Gene Ontology (GO) analysis. The analysis revealed that several candidate target genes were predominantly enriched in pathways related to development, cell communication, and signaling pathways (Figure 2G). Notably, the most enriched top pathway was tissue development, which included 14 candidate target genes. Among these, the top two candidate target genes, *ecdysone receptor* (*EcR*) and *chitinase 10* (*Cht10*), exhibited significantly reduced m^6^A enrichment at the late stage compared to the early stage, along with > 3‐fold decreased transcript levels in ds*Mettl3*‐injected nymphs relative to ds*GFP*‐injected controls (Figure 2G,H; Figure S3C, Supporting Information). Additionally, several m^6^A regulatory components, including two readers (*Hnrnp* and *eIF3‐S6*), an eraser (*Alkbh5*), and rRNA methyltransferases (*Mettl5* and *Zcchc4*), were also found to be m^6^A‐modified and showed similar reduction patterns in both m^6^A enrichment (late vs early stage) and transcript abundance (ds*Mettl3* vs ds*GFP* groups) (Figure S3C, Supporting Information). We further validated the m^6^A level of *EcR* and *Cht10* using MeRIP‐qPCR and assessed their expression levels after knockdown of *Mettl3* by qPCR. The results showed that *EcR* and *Cht10* have higher m^6^A levels in the MeRIP integuments than in the IgG group (*t* = 4.766, *p* < 0.001 for *EcR*, *t* = 16.44, *p* < 0.001 for *Cht10*, Figure 2H). The expression levels of *EcR* and *Cht10* were significantly down‐regulated when *Mettl3* was knocked down in locusts (*t* = 4.490, *p* < 0.01 for *EcR*, *t* = 3.096, *p* < 0.01 for *Cht10*, Figure 2H). Ecdysone receptor (EcR), a member of the nuclear receptor family, is well known to meditate the signaling of the steroid hormone ecdysone in insects.^[^ ^33^, ^34^ ^]^ Chitinase, on the other hand, catalyzes the degradation of chitin in insects.^[^ ^35^, ^36^ ^]^ These promising results led us to investigate whether the dynamic changes in m^6^A levels of these two target genes at different stages of each instar nymphs were associated with molting events.

### 2.3 m^6^A Reader eIF3‐S6 Binds with *EcR/Cht10* by Recognizing m^6^A Marks for Molting

**Figure 3:**
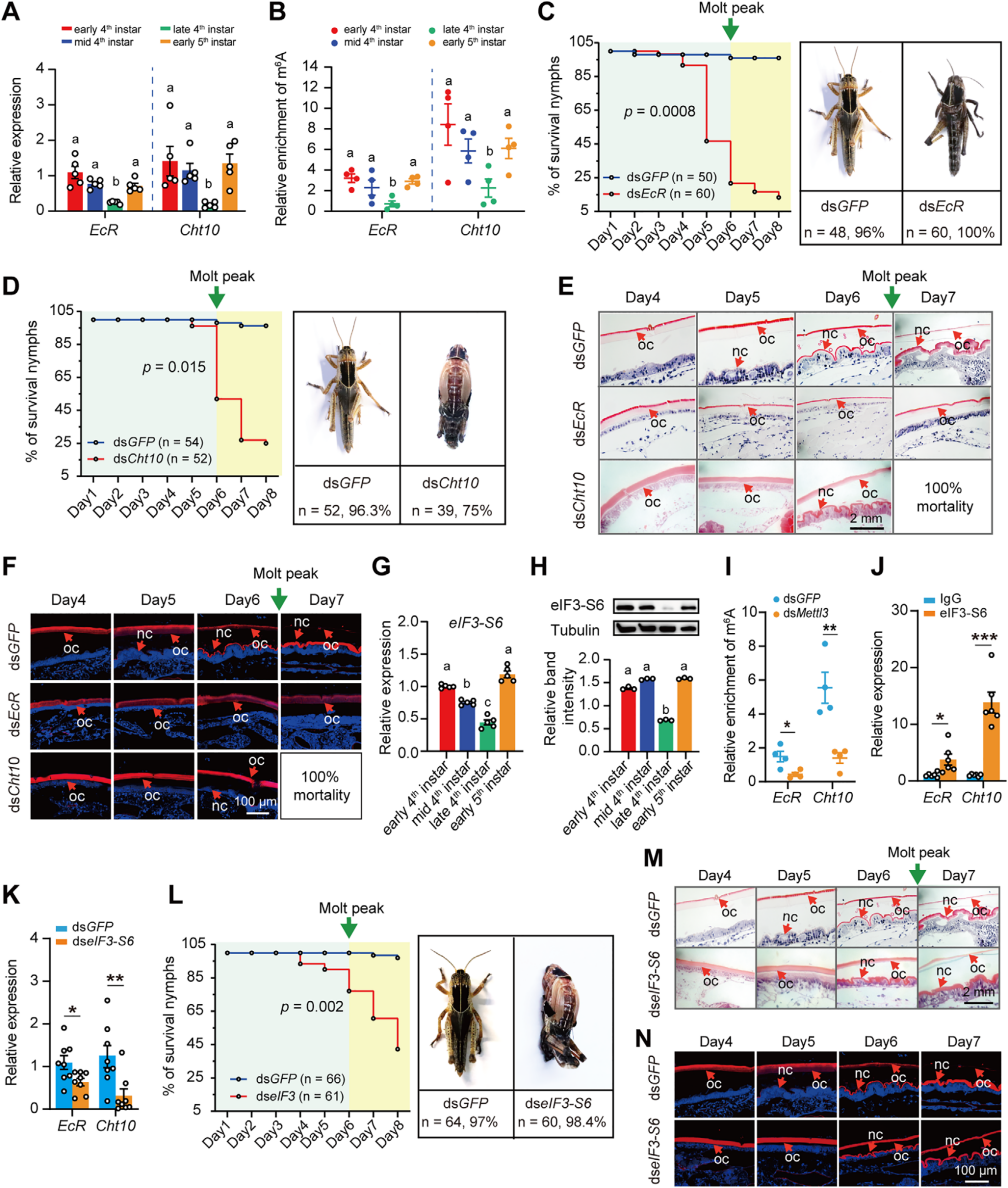
m^6^A reader eIF3‐S6 binds with *EcR/Cht10* by recognizing m^6^A marks for molting. A) The expression levels of *EcR* and *Cht10* were determined in the integuments from early‐, mid‐, and late‐stage of the 4^th^ instar nymph and early‐stage of the 5^th^ instar nymph by qPCR. B) m^6^A‐IP‐qPCR analysis of m^6^A level for *EcR* and *Cht10* in the integuments from early‐, mid‐, and late‐stage of the 4^th^ instar nymph and early‐stage of the 5^th^ instar nymph. C,D) The effects of silencing *EcR* (C) and *Cht10* (D) on the mortality and molting process of locusts. The green arrow indicates the time point for molting. E,F) The effects of silencing *EcR* (E) and *Cht10* (F) on the metabolism of both new and old cuticles were assessed through Hematoxylin and eosin staining (E) and chitin staining (F). OC, old cuticle, NC, new cuticle. G,H) Relative expression levels of eIF3‐S6 in the integument of early‐, mid‐, and late‐stage of the 4^th^ instar nymph and early‐stage of the 5^th^ instar nymph by qPCR (G) and western blot (H). I) m^6^A‐IP‐qPCR analysis of m^6^A level for *EcR* and *Cht10* after knockdown of *Mettl3*. J) RIP‐qPCR showing enrichment of *EcR* and *Cht10* mRNA by the eIF3‐S6 antibody compared to IgG. K) The mRNA expression levels of *EcR* and *Cht10* after knockdown of *eIF3‐S6* in the locust integument. L) The effects of silencing *eIF3‐S6* on the mortality and molting of locusts, with the green arrow indicating the time for molting. M,N) The effect of silencing *eIF3‐S6* on the metabolism of new and old cuticle. Hematoxylin and eosin staining (M) and chitin staining (N) of the integument were determined in the locusts. Different letters indicate statistically significant differences between groups using one‐way ANOVA (Tukey's multiple comparisons test, *p* < 0.05) (A, B, G, and H). *P* values in survival curve were determined by the Kaplan–Meier statistical analysis (C, D, L). *P* values in MeRIP‐qPCR, RIP‐qPCR, and qPCR were determined by a two‐tailed unpaired *t*‐test (I, J, K). Data are presented as mean ± SEM. ^*^ *p* <0.05, ^**^ *p* < 0.01, ^***^ *p* < 0.001.

To confirm whether *EcR* and *Cht10* are regulated by m^6^A modification during molting, we first performed a comprehensive analysis combined their expression patterns and m^6^A modification levels across the molting process. We observed a significant correlation between a sharp fall in *EcR* and *Cht10* expressions and a drop in their m^6^A modification levels during nymphs molting (**Figure** **3**A,B).

To verify the function of *EcR* and *Cht10* in molting, we performed RNAi knockdown of *EcR* or *Cht10* into locusts (Figure S5, Supporting Information). During molting, locusts injected with ds*EcR* or ds*Cht10* displayed a clear molting defect. Specially, all 60 nymphs injected with ds*EcR*, died (100%) during the molting process from the 4^th^ to 5^th^ instar, compared to only 4% (2 out of 50) of mortality in the control group (Figure 3C). Similarly, 75% of nymphs died after injection of ds*Cht10*, relative to only 3.7% of mortality observed in the control group (Figure 3D).

We further examined the impact of *EcR* and *Cht10* knockdown on integument structure using hematoxylin and eosin staining, as well as chitin staining (Figure 3E,F). Knockdown of *EcR* not only severely reduced chitin levels in the newly formed cuticle, reflecting impaired synthesis, but also prevented chitin degradation in the old cuticle. In contrast, *Cht10* knockdown inhibited the degradation of old cuticle, the chitin of which was not diminished compared with that of the controls (Figure 3E,F). Thus, *EcR* and *Cht10* are integral to the molting process in locusts, mediating the metabolism of both new and old cuticles.

To explore the relationship between m^6^A and ecdysone signaling target genes, we identified six reader genes in the locust genome, including YTH homologs (*Ythdc1* and *Ythdf2*), eIF3 homolog (*eIF3‐S6*), HNRNP homologs (*Hnrnp* and *Hnrnpc*) and IGF2BP homolog (*Igf2bp3*) (Figure 3G; Figure S4, Supporting Information). We then analyzed the expression patterns of these readers in relation to *EcR* and *Cht10*. Notably, only *eIF3‐S6* showed a similar dynamic wave‐like pattern to *EcR and Cht10*, with peak expression at the early instar and bottom levels at the late instar at both mRNA and protein levels (Figure 3A,G,H). In contrast, the other 5 readers exhibited inconsistent patterns to *EcR and Cht10* (Figure 3A; Figure S4, Supporting Information). The results imply a potential binding relationship between the reader *eIF3‐S6* and the molting targeting genes *EcR and Cht10*.

To determine whether eIF3‐S6 binds to *EcR and Cht10* by recognizing m^6^A marks on mRNAs, we measured m^6^A levels of *EcR* and *Cht10* after *Mettl3* knockdown by MeRIP qPCR. The m^6^A level of *EcR* and *Cht10* significantly decreased in the locusts that underwent *Mettl3* knockdown, compared to locusts that received ds*GFP* injection (*t* = 3.168, *p* < 0.05 for *EcR*; *t* = 4.261, *p* < 0.01 for *Cht10*; Figure 3I). Additionally, we performed an RNA immunoprecipitation (RIP) assay using an antibody against the eIF3‐S6 protein in locust integument (Figure 3J). Both *EcR* and *Cht10* were significantly enriched in the eIF3‐S6‐immunoprecipitated RNAs compared to those from IgG controls (*t* = 2.647, *p* < 0.05 for *EcR*; *t* = 6.986, *p* < 0.0001 for *Cht10*; Figure 3J). Thus, eIF3‐S6 can directly bind to m^6^A marks of *EcR* and *Cht10* in the locust integument.

To determine the effects of eIF3‐S6 on its target genes *EcR* and *Cht10* in molting, we quantified mRNA expression levels of *EcR* and *Cht10* after the knockdown of *eIF3‐S6* in locust integument (Figure 3K; Figure S5, Supporting Information). Injection of ds*eIF3‐S6* led to significant decreases in *EcR* and *Cht10* mRNA levels compared to those in control locusts (*t* = 2.469, *p* < 0.05 for *EcR*; *t* = 3.029, *p* < 0.01 for *Cht10*; Figure 3K). To determine whether knockdown of *eIF3‐S6* causes abnormal molting, we injected dsRNA targeting *eIF3‐S6* into locusts. The nymphs displayed *EcR*‐ and *Cht10*‐like molting defects (Figure 3L), with 98.4% mortality from molting arrest. Abnormal individuals retained old cuticle fragments on legs, resulting from impaired chitin degradation that caused old cuticular thickening and mechanical shedding obstruction (Figure 3L–N).

### 2.4 eIF3‐S6 Binds with *EcR/Cht10* by Recognizing *EcR* A^335^ and *Cht10* A^7589^

**Figure 4:**
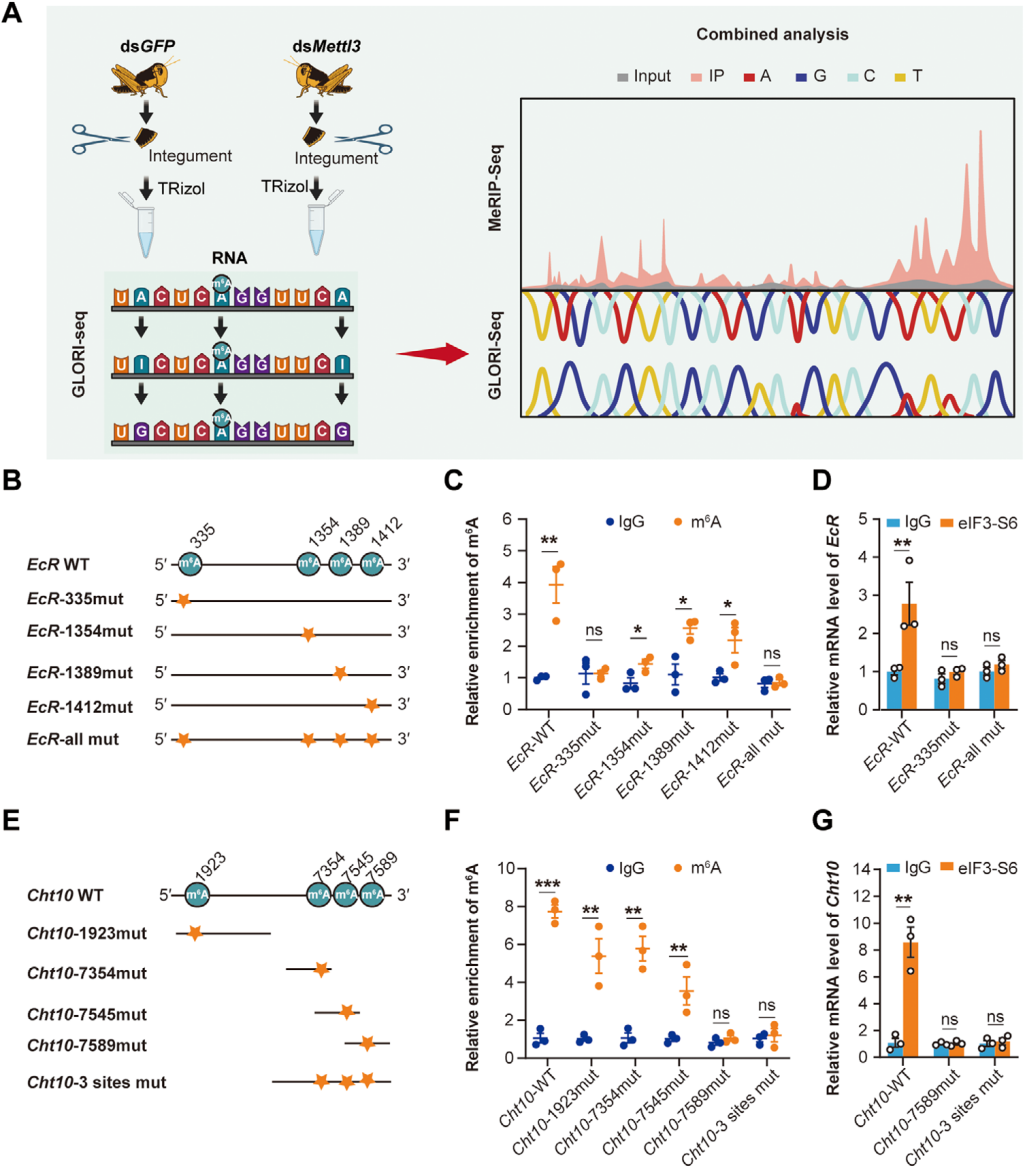
eIF3‐S6 binds with *EcR/Cht10* by recognizing *EcR* A^335^ and *Cht10* A^7589^. A) The distribution of m^6^A sites across the transcriptome from the schematic of MeRIP‐seq and GLORI‐seq analysis. B) The four sites (A^335^, A^1354^, A^1389^, and A^1412^) of *EcR* were identified as potential methylation sites. C) The four m^6^A sites (A^335^, A^1354^, A^1389^, and A^1412^) of *EcR* were verified using *EcR* mutants (*EcR* A^335^C, A^1354^C, A^1389^C, A^1412^C, and a quadruple mutant) or *EcR* WT transfection into S2 cells by m^6^A‐IP‐qPCR assays. D) eIF3‐S6 exhibited strong binding affinity with the *EcR* A^335^ site by eIF3‐S6 RIP‐qPCR when transfection of *EcR* WT and *EcR* mutants into S2 cells. E) The four sites (A^1923^, A^7354^, A^7545^, and A^7589^) of *Cht10* were identified as potential methylation sites. F) The four m^6^A sites (A^1923^, A^7354^, A^7545^, and A^7589^) of *Cht10* were verified using *Cht10* mutants (*Cht10* A^1923^C, A^7354^C, A^7545^C, A^7589^C or a quadruple mutant) or *Cht10* WT transfection into S2 cell by m^6^A‐IP‐qPCR assays. G) eIF3‐S6 exhibited strong binding affinity with *Cht10* A^7589^ site by eIF3‐S6 RIP‐qPCR when transfection of *Cht10* WT and *Cht10* mutants into S2 cells. *P* values in MeRIP‐qPCR and RIP‐qPCR were determined by a two‐tailed unpaired *t*‐test (C, D, F and G). Data are presented as mean ± SEM. ^*^ *p* < 0.05, ^**^ *p* < 0.01, ^***^ *p* < 0.001, ns, not significant.

We aimed to point the exact locations of m^6^A sites by employing GLORI (Global locating of RNA m^6^A at single‐nucleotide resolution) sequencing to map the m^6^A sites in *EcR* and *Cht10* following knockdown of *Mettl3*. This approach revealed four potential methylation sites in *EcR* (A^335^, A^1354^, A^1389^, and A^1412^) and four sites in *Cht10* (A^1923^, A^7354^, A^7545^, and A^7589^) (**Figure** **4**A,B,E). To verify the reliability of these m^6^A sites, we generated *EcR* mutants (*EcR* A^335^C, A^1354^C, A^1389^C, A^1412^C and a quadruple mutant, Figure S6, Supporting Information) and *Cht10* mutants (*Cht10* A^1923^C, A^7354^C, A^7545^C, A^7589^C or a quadruple mutant, Figure S6, Supporting Information) to analyze their effects on m^6^A level (Figure 4C,F) and eIF3‐S6 binding capacity (Figure 4D,G). The MeRIP‐qPCR data showed that *EcR* mutants A^1354^C, A^1389^C, and A^1412^C as well as *Cht10* mutants A^1923^C, A^7354^C, and A^7545^C, were robustly enriched by an m^6^A‐specific antibody, compared to the control IgG antibody. In contrast, enrichment was absent in both *EcR* (A^335^C and quadruple) and *Cht10* (A^7589^C and quadruple) mutants (Figure 4C,F). This suggests that m^6^A modification was repressed in these mutants due to the mutation at *EcR* A^335^ site and *Cht10* A^7589^ sites, rather than the mutations at *EcR* A^1354^C, A^1389^C, A^1412^C as well as *Cht10* A^1923^C, A^7354^C, A^7545^C sites. Thus, *EcR* A^335^ and *Cht10* A^7589^ are indeed m^6^A‐modified sites.

Furthermore, we examined eIF3‐S6 binding affinity to transfected cellular wild‐type and mutant RNAs. The eIF3‐S6 RIP experiment showed that *EcR* A^335^C mutant and *EcR* quadruple mutant as well as *Cht10* A^7589^C and *Cht10* quadruple mutant, exhibited diminished eIF3‐S6 binding to mutant RNA compared to wild‐type RNA (Figure 4D,G). These results indicate that mutagenesis of the m^6^A‐modified sites diminished eIF3‐S6 recognition and that eIF3‐S6 bound to *EcR* and *Cht10* by recognizing the *EcR* A^335^ and *Cht10* A^7589^ sites.

### 2.5 eIF3‐S6 Undergoes Phase Separation in Molting

**Figure 5:**
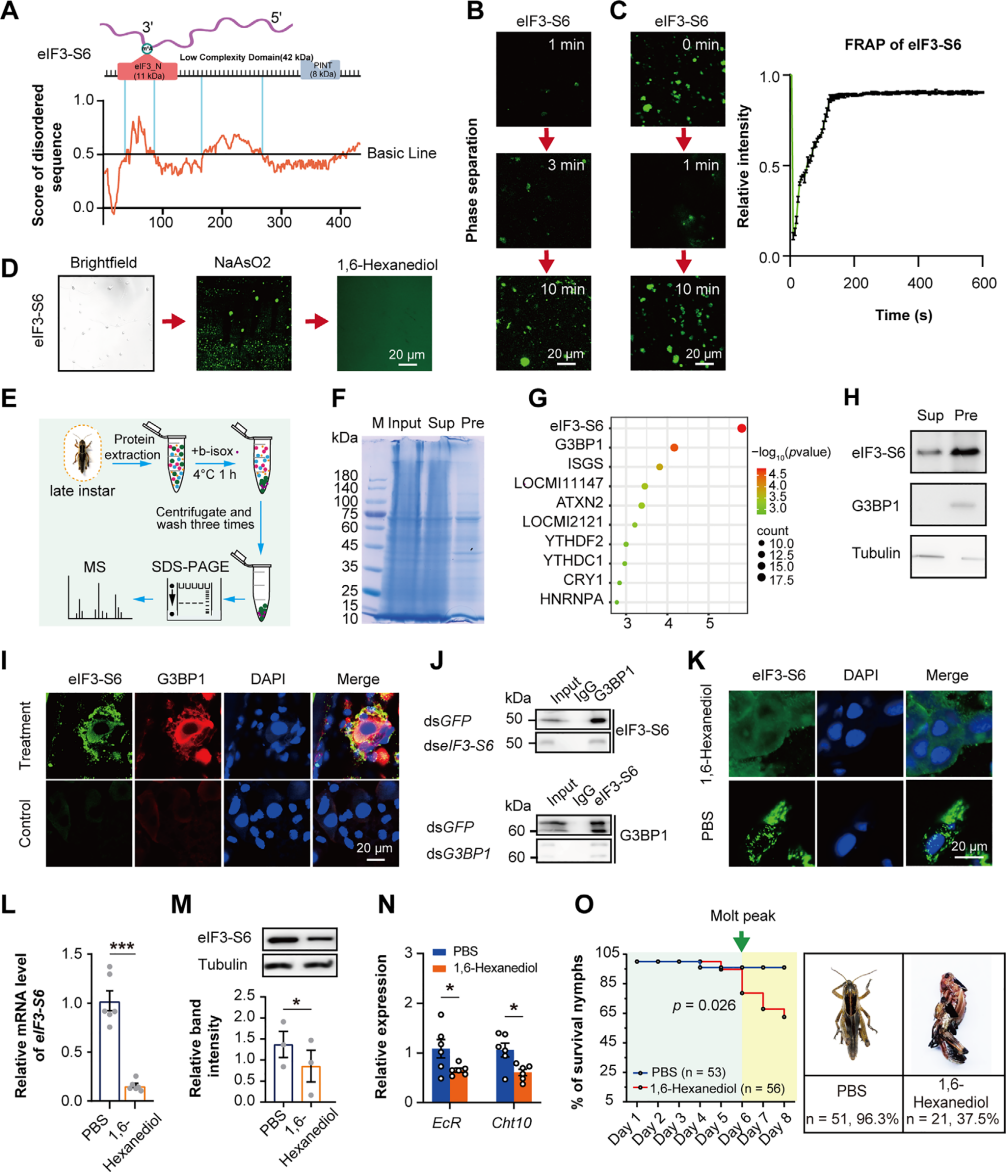
eIF3‐S6 undergoes phase separation in molting. A) The prion‐like domain disorder sequence was generated using the PLAAC (Prion‐like amino acid composition) tool. The low complexity domain is a ≈42 kDa region. The ≈11 kDa eIF3‐N domain shows binding affinity to m^6^A. B) Phase separation assays in vitro were performed to detect the formation of eIF3‐S6 droplets. The small droplets of purified recombinant eIF3‐S6 underwent fusion to form larger ones over time. C) Fluorescence recovery after photo‐bleaching (FRAP) assays were used to detect the fluorescence recovery of eIF3‐S6 droplets. The changes in the fluorescence intensity of the droplets post‐photobleaching were plotted over time, with the curve representing the mean fluorescence intensity across distinct droplets (n = 4). D) The phase separation of eIF3‐S6 was enhanced by increasing of salt (NaCl) and inhibited by 1, 6‐Hexanediol. E) The procedure of b‐isox precipitation and mass spectrometry analysis. F) The integuments lysate from the molt stage was subjected to precipitation by b‐isox. Coomassie brilliant blue staining of an SDS‐polyacrylamide gel in total, supernatant, and b‐isox precipitation samples, as analyzed by western blotting. Size markers are labeled left of the gel. G) Ten proteins were identified from mass spectrometry analysis on b‐isox precipitation of integuments from the late stage of the 4^th^ instar nymphs. H) Relative protein levels (n = 3) of both eIF3‐S6 and G3BP1 in b‐isox supernatant and precipitation from integuments of the late stage of the 4^th^ instar nymphs. I) eIF3‐S6 and G3BP1 colocalized within the same condensed droplets in the integument cells of locusts through an immunohistochemistry assay. J) The interaction between eIF3‐S6 and G3BP1 in locust integuments was confirmed by co‐immunoprecipitation assays. K) The distribution of eIF3‐S6 condensation droplets in the primary cells of the locust integument after the injection of 1, 6‐Hexanediol by immunohistochemistry assays, injection of PBS is used as the control. L–N) The expression levels of *eIF3‐S6* (L for mRNA level, M for protein level) and *EcR*/*Cht10* (N) were assessed in the locust integuments after the injection of 1, 6‐Hexanediol by qPCR and western blot. O) The effects of 1, 6‐Hexanediol on locust mortality were analyzed after injection. The molting phenotype showed that the nymphs injected with 1, 6‐Hexanediol exhibited abnormal molting. *P* values were determined by a two‐tailed unpaired *t*‐test (L, M, and N). *P* values in survival curve were determined by the Kaplan–Meier statistical analysis (O). Data are presented as mean ± SEM. ^*^ *p* <0.05, ^***^ *p* < 0.001.

To understand the impact of m^6^A on the expression of molting‐related genes *EcR* and *Cht10*, we investigated the biochemical properties of m^6^A‐binding protein eIF3‐S6. This protein comprises an 11 kDa eIF3_N domain for m^6^A binding, and a 42 kDa low‐complexity domain with prion‐like domains based on computational Prion‐Like Amino Acid Composition (PLAAC) analysis (**Figure** **5**A; Figure S7A, Supporting Information). We hypothesized that low‐complexity sequences might form liquid droplets due to phase separation, as previously reported (5,6). To test this hypothesis, we purified full‐length eIF3‐S6 from locusts (Figure S7B, Supporting Information), and observed the formation of spherical droplets that underwent time‐dependent coalescence from small to large sizes under phase contrast microscopy (Figure 5B). Following photobleaching (FRAP), eIF3‐S6 droplets showed dynamic fluorescence recovery, reaching near‐initial signal intensity within ≈150 s (Figure 5C). The formation of eIF3‐S6 droplets was enhanced by NaAsO2 and dampened by 1, 6‐Hexanediol, a phase separation inhibitor (Figure 5D). Overall, these data indicated that eIF3‐S6 exhibits characteristics of liquid‐liquid phase separation (LLPS) in vitro.

To confirm whether phase separation occurs during molting, we identified potential phase‐separation proteins in the integument of molting locusts using b‐isox precipitations (Figure 5E). Mass spectrometry analysis of b‐isox precipitates identified 10 proteins (Figure 5F), including eIF3‐S6, which showed significantly enriched (*p* < 0.001, fold change = 15.3, Figure 5G). Other proteins known to trigger and undergo LLPS, such as G3BP1, Ythdf2, and Ythdc1,^[^ ^37^, ^38^, ^39^ ^]^ were also enriched (Figure 5G), implying an important role for phase separation in molting. To confirm whether Ythdf2 and Ythdc1, as the m^6^A readers,^[^ ^28^, ^40^ ^]^ were responsible for the molting, we monitored locusts molting after ds*Ythdf2* and ds*Ythdc1* injection, respectively (Figure S8A,C, Supporting Information). The nymphs molted normally, with mortality rates of 12.7% for ds*Ythdf2* and 8.8% for ds*Ythdc1*, compared to 3.6% for ds*GFP*‐injected controls (Figure S8B,D, Supporting Information). We furtherly investigated whether eIF3‐S6 undergoes phase separation to form condensates in the locust integument in molting. G3BP1, which acts as a molecular marker of ribonucleoprotein (RNP) granule and can trigger RNA‐dependent LLPS.^[^ ^39^ ^]^ We next examined whether eIF3‐S6 and G3BP1 colocalize in locust integuments. As expected, both eIF3‐S6 and G3BP1 were observed in the same condensed cytoplasmic droplets within integument cells (Figure 5H,I). Furthermore, co‐immunoprecipitation assays confirmed a direct interaction between eIF3‐S6 and G3BP1 in locust integuments (Figure 5J). Thus, the direct interaction between eIF3‐S6 and G3BP1 in cytoplasmic condensates was mediated through LLPS.

To further verify the function of LLPS in molting, we injected 1, 6‐Hexanediol, into locusts at the pre‐molt stage and observed a significant reduction of eIF3‐S6 condensation droplets in the integument (Figure 5K). Since eIF3‐S6 can bind *EcR* and *Cht10* through recognizing m^6^A marks, we assessed their expression levels after injection of 1, 6‐Hexanediol, and found a significant decrease (*t* = 8.334, *p* < 0.0001 for *eIF3‐S6* mRNA level; *t* = 6.263, *p* < 0.05 for *eIF3‐S6* protein level; *t* = 2.269, *p* < 0.05 for *EcR*; *t* = 2.932, *p* < 0.05 for *Cht10*) (Figure 5L–N). These results suggest that eIF3‐S6 undergoes LLPS by binding with *EcR* and *Cht10* during molting.

Locusts treated with 1,6‐hexanediol exhibited significantly higher rates of molting abnormalities and mortality (37.5%) compared to controls (3.7%) (Figure 5O). Therefore, the phase separation‐mediated by eIF3‐S6 condensates with *EcR* and *Cht10* plays a critical role in regulating molting.

### 2.6 eIF3‐S6 Binds with m^6^A*‐EcR*/*Cht10* to form Droplet Granules for Protecting the Target mRNAs Stability

**Figure 6:**
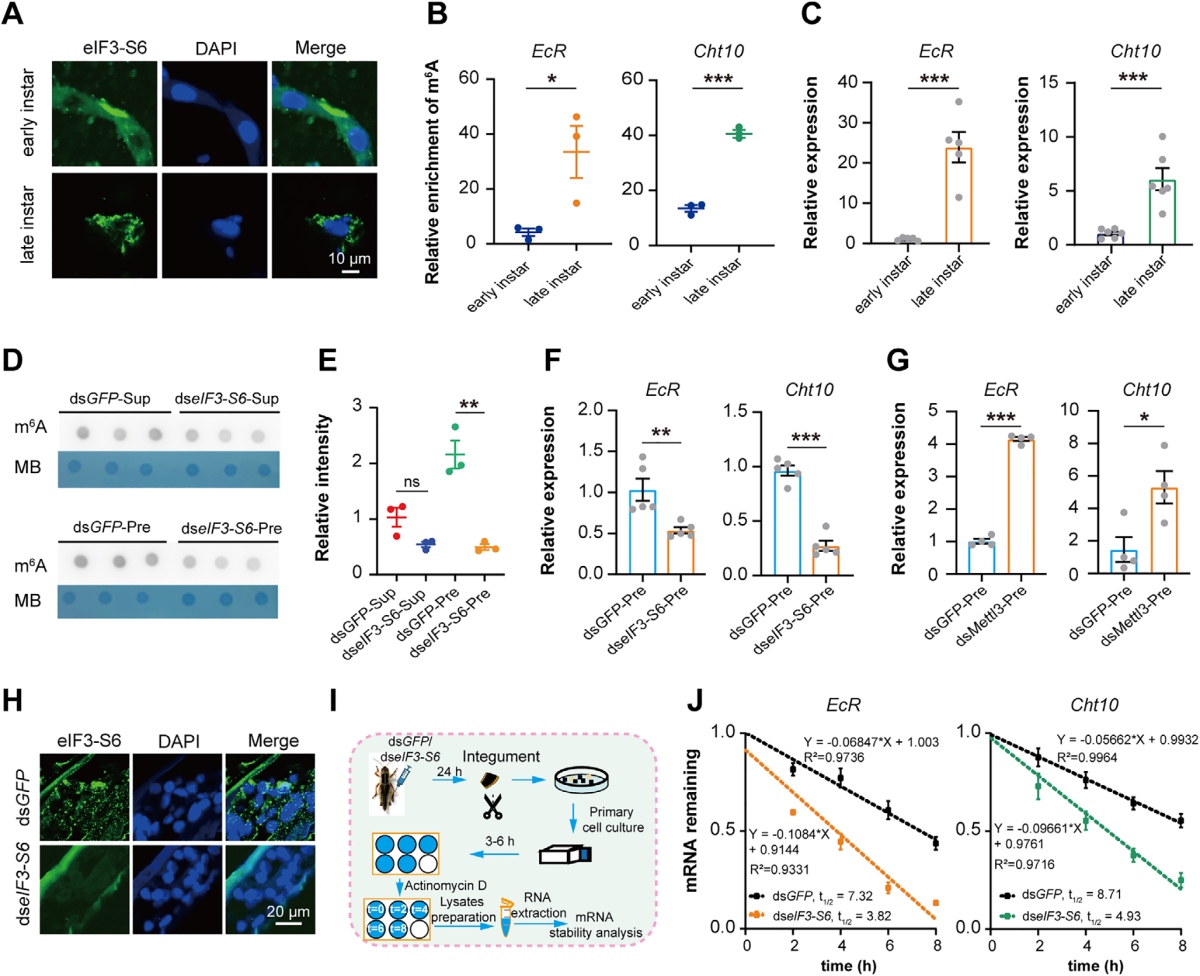
eIF3‐S6 binds with m^6^A*‐EcR*/*Cht10* to form droplet granules for protecting the target mRNAs stability. A) eIF3‐S6 formed spherical condensates that displayed a strong fusion signal in the primary integument cells of the late instar, but markedly diminished in those of the early instar by immunohistochemistry assay. B) m^6^A‐IP‐qPCR analysis of m^6^A level for *EcR* and *Cht10* in both early instar and late instar of locusts after b‐isox precipitation. C) The expression levels of *EcR* and *Cht10* were evaluated in both early instar and late instar by qPCR after b‐isox precipitation. D,E) The total RNA m^6^A levels in the b‐isox precipitation or supernatant of the ds*eIF3‐S6*‐injected locusts. The ds*GFP*‐injected locusts served as controls (D), and dot blot bands were quantified using densitometry (E). F) The mRNA expression levels of *EcR* and *Cht10* in the b‐isox precipitation of the ds*eIF3‐S6* and ds*GFP*‐injected locusts by qPCR. G) The b‐isox precipitation‐qPCR analysis of expression levels for *EcR* and *Cht10* in the condensate droplets of the locust integuments after RNAi *Mettl3*. H) The distribution of eIF3‐S6 condensation droplets in the locust integument following *eIF3‐S6*‐knockdown by immunohistochemistry assays. I) The procedure of RNA stability assay. J) The half‐lives of *EcR* and *Cht10* mRNA in the primary cells of locusts at the late instar following *eIF3‐S6*‐knockdown. *P* values were determined by a two‐tailed unpaired *t*‐test (B, C, E and F). Data are presented as mean ± SEM. ^*^ *p* < 0.05, ^**^ *p* < 0.01, ^***^ *p* < 0.001. ns, not significant.

To examine the role of eIF3‐S6 in molting through phase separation dynamics, we isolated integument primary cells and compared eIF3‐S6 condensates in early (pre‐molt stage) and late instars (molt stage). The results showed that eIF3‐S6 formed fusion condensates with intense signals in late‐ but not early‐instar integument cells (**Figure** **6**A), indicating molting‐stage‐specific phase separation is required for normal molting.

Given our findings that eIF3‐S6 recognizes and binds m^6^A marks on both *EcR* and *Cht10* in locust integuments, we next asked whether these m^6^A‐marked transcripts were specifically recruited into eIF3‐S6 condensates at the critical late instar period. We employed b‐isox precipitation analysis and anti‐m^6^A IP‐qPCR (MeRIP‐qPCR) to compare m^6^A levels of *EcR* and *Cht10* transcripts across early and late instars. The results showed that significantly higher m^6^A enrichment of both transcripts in late‐instar condensates compared to early‐instar counterparts (*t* = 3.038, *p* < 0.05 for *EcR*, *t* = 14.25, *p* < 0.0001 for *Cht10*, Figure 6B). Consistently, b‐isox precipitation followed by qPCR analysis revealed significantly higher expression levels of both *EcR* and *Cht10* transcripts in late‐instar condensates than those in early‐instar condensates (*t* = 2.644, *p* < 0.05 for *EcR*, *t* = 4.815, *p* < 0.0001 for *Cht10*, Figure 6C). Thus, the interaction between eIF3‐S6 and m^6^A‐modified *EcR*/*Cht10* transcripts drives phase separation within biomolecular condensates during locust molting.

To confirm the functional interdependence between eIF3‐S6 and m^6^A‐modified *EcR*/*Cht10* transcripts in condensates, we performed eIF3‐S6 knockdown in late‐instar locusts and measured m^6^A levels in condensate‐enriched mRNA. The m^6^A levels revealed a 77% reduction in condensate‐associated RNAs (b‐isox precipitation) from ds*eIF3‐S6*‐injected locusts compared to ds*GFP* controls (*t* = 6.498, *p* < 0.01). In contrast, m^6^A levels in the non‐condensate fraction (b‐isox supernatant) showed no significant difference between ds*eIF3‐S6* and ds*GFP* groups (*t* = 6.303, *p* = 0.0032). (Figure 6D,E). Furthermore, mRNA levels of *EcR* and *Cht10* in b‐isox‐isolated condensates were significantly reduced by 47% (*t* = 3.507, *p* < 0.01) and 72% (*t* = 10.46, *p* < 0.001), respectively, in ds*eIF3‐S6*‐injected locusts compared to ds*GFP* controls (Figure 6F). Consistently, RNAi knockdown of *Mettl3* in locusts significantly increases *EcR* and *Cht10* expression levels within these granules (Figure 6G), revealing a critical link between m^6^A modification and phase separation. Immunofluorescence analysis revealed a marked decrease in eIF3‐S6‐positive condensates in the integument following *eIF3‐S6* knockdown (Figure 6H). RNA stability assays revealed that the half‐lives of *EcR* and *Cht10* mRNAs were significantly reduced in primary integument cells from *eIF3‐S6*‐knockdown locusts at the late‐instar (Figure 6I,J), demonstrating that eIF3‐S6‐containing condensates stabilize these transcripts during molting. All together, these data indicated that eIF3‐S6 binds to m^6^A*‐EcR*/*Cht10* in a mutually dependent manner to form condensates that protect target mRNA stability.

### 2.7 Disturbance of m^6^A Modifications Leads to Decreased Phase Separation and Molting Failure

**Figure 7:**
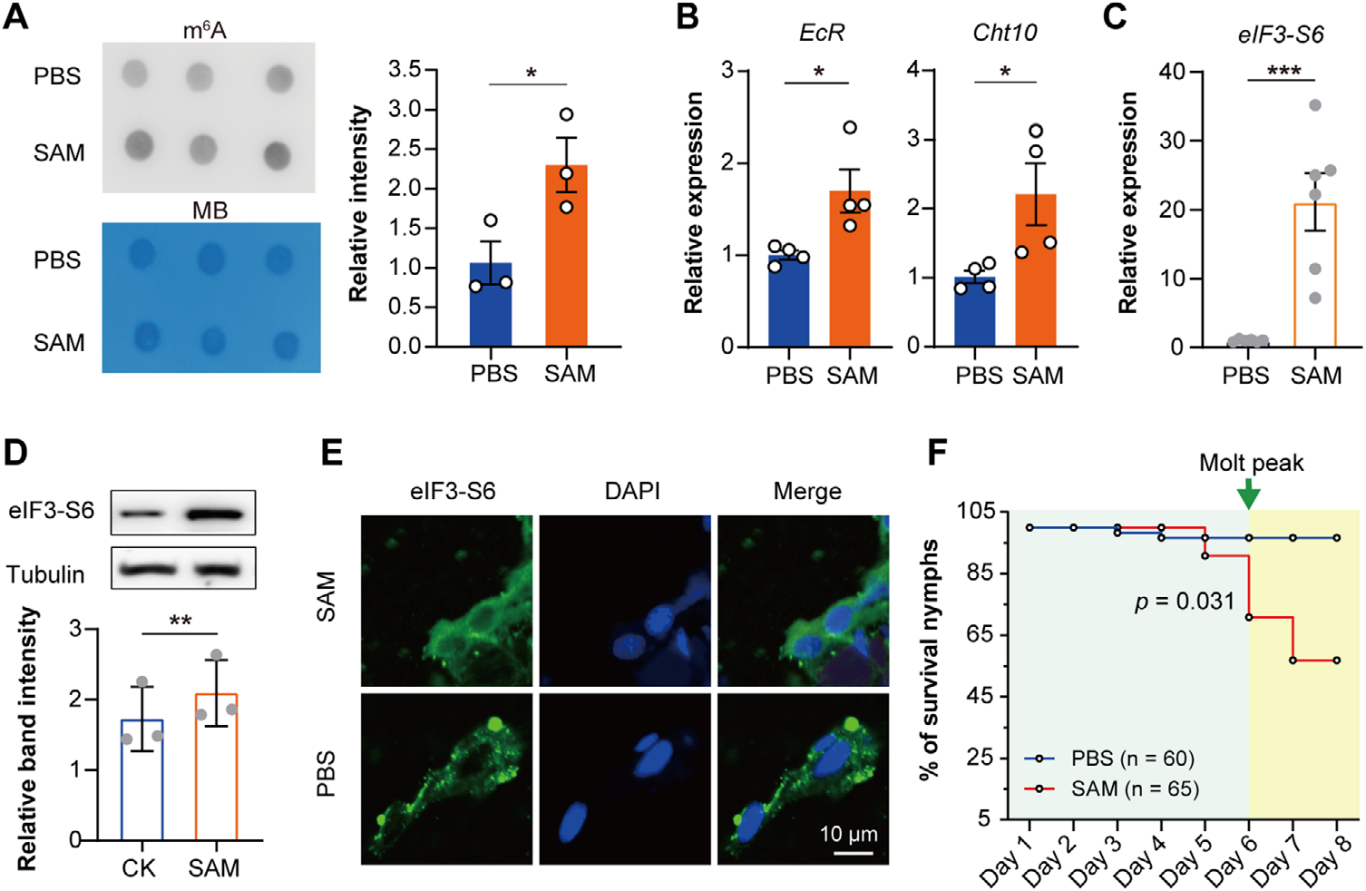
Decreased m^6^A modifications promote phase separation associated with the locust molting. A) The RNA m^6^A levels from locusts at the late‐stage of the 4^th^ instar nymphs after the injection of S‐adenosylmethionine (SAM). Dot blot bands were quantified using densitometry. B) The expression levels of *EcR* and *Cht10* in the late‐stage of the 4^th^ instar nymphs after the injection of SAM by qPCR. C,D) The expression levels of eIF3‐S6 in the late‐stage of the 4^th^ instar nymphs after the injection of SAM by qPCR (C) or western blot (D). E) The distribution of eIF3‐S6 condensation droplets in the locust integument following SAM injection or PBS injection (as the control) by immunohistochemistry assays. F) The effects of SAM on locust mortality. *P* values were determined by a two‐tailed unpaired *t*‐test (A–D). *P* values in survival curve were determined by the Kaplan–Meier statistical analysis (F). Data are presented as mean ± SEM. ^*^ *p* < 0.05, ^**^ *p* < 0.01, ^***^ *p* < 0.001.

During the molting stage, the minimal levels of m^6^A modification on mRNAs coincided with eIF3‐S6 phase separation. We suspect that disturbing m^6^A levels could influence LLPS. To test this, we injected S‐adenosylmethionine (SAM), a universal methyl donor, into locusts at the mid‐instar stage, and we observed a significant increase in m^6^A levels of mRNAs in the late‐instar stage (*t* = 2.838, *p* < 0.05, **Figure** **7**A). Moreover, SAM injection not only induced an elevated expression of *EcR* and *Cht10* (*t* = 2.900, *p* < 0.05 for *EcR*; *t* = 2.606, *p* < 0.05 for *Cht10*; Figure 7B), but also promoted eIF3‐S6 levels in the integuments of locusts both at the mRNA (*t* = 4.814, *p* < 0.001; Figure 7C) and protein levels (*t* = 35.36, *p* < 0.001; Figure 7D).

Upon examining eIF3‐S6 localization in the integument after SAM injection, we observed that eIF3‐S6 remained diffusely cytoplasmic and failed to form distinct granule droplets. In contrast, PBS‐injected controls (with low m^6^A levels) exhibited clear droplet‐like eIF3‐S6 granules (Figure 7E). Thus, eIF3‐S6 granule formation depends on the maintenance of low m^6^A levels during molting.

To determine whether the loss of droplet granules in SAM‐injected locusts affects molting, we monitored nymphs until the molt stage. These individuals exhibited abnormal molting and failed to complete ecdysis, resulting in a 54% mortality rate (Figure 7F). Our findings demonstrate that m^6^A‐dependent regulation in the integument is essential for orchestrating phase separation during locust molting.

## 3 Discussion

**Figure 8:**
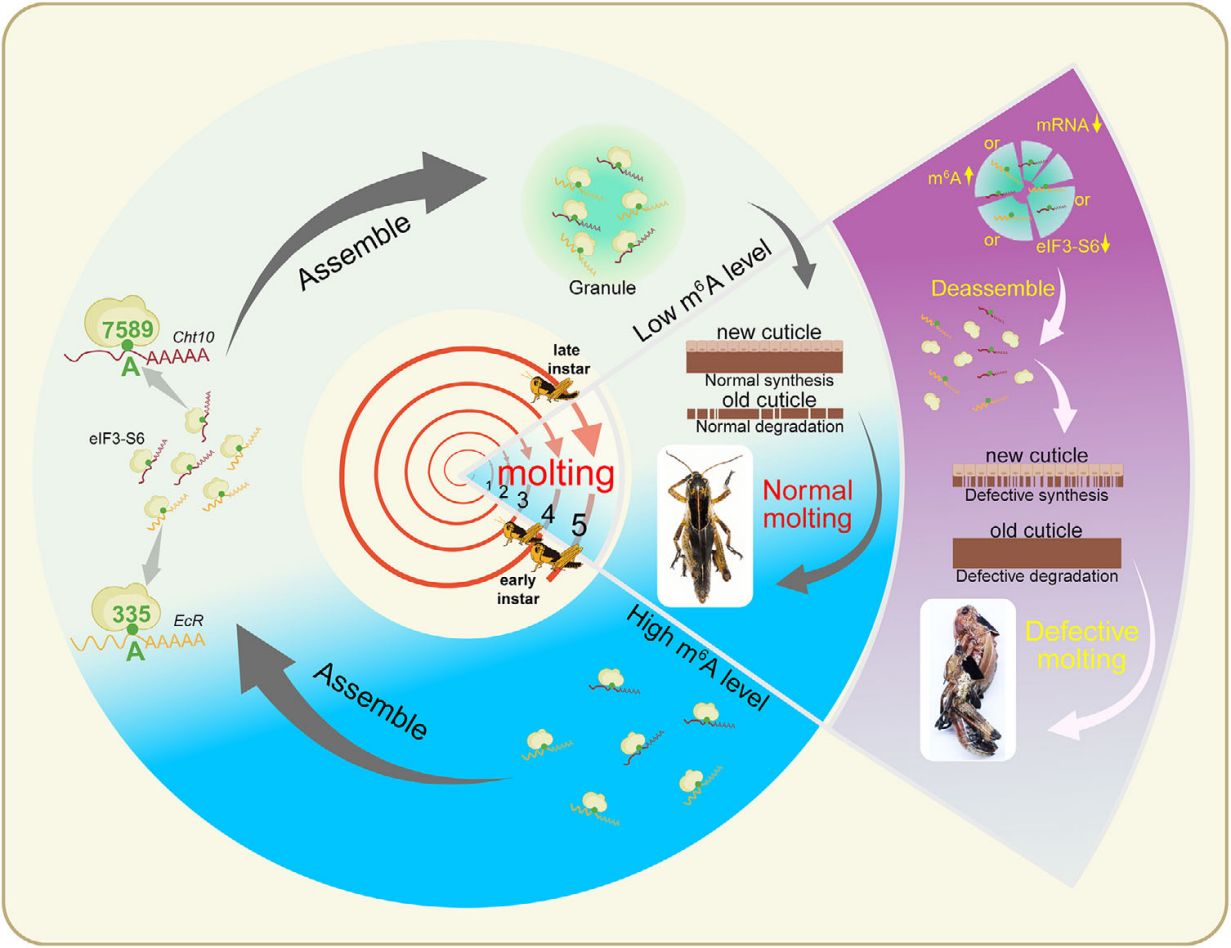
Model of a mechanism for m^6^A modification‐instructed LLPS as a key driver in locusts molting. eIF3‐S6, as a key m^6^A reader in integument, recruits two key m^6^A‐modified *EcR* and *Cht10* to undergo dynamic phase separation, thus promoting *EcR* and *Cht10* mRNA stability for normal molting. In the pre‐molt stage, m^6^A modification of molting‐responsive genes coordinates their expression in a timely and dynamic fashion. However, in the molt stage, the molting‐responsive *EcR* and *Cht10* undergo degradation with the decrease of their m^6^A levels. Consequently, the eIF3‐S6 binding with *EcR* A^335^ /*Cht10* A^7589^ forms condensation droplets, which protect *EcR* and *Cht10* from excessive degradation, thereby ensuring normal molting. Knockdown of each droplet member or disruption of m^6^A levels leads to the absent of phase separation, leading to abnormal molting.

Insect molting serves as a model for understanding how multi‐regulatory mechanisms coordinate to ensure the precise temporal expression of molting‐responsive genes during tissue degradation and remodeling. Here, we investigated the dynamic interplay between molting‐responsive RNA transcription, m^6^A modification levels, and m^6^A reader phase separation in response to molting signals. Our findings demonstrate that during the pre‐molt stage, m^6^A modification dynamically coordinates the temporal expression of molting‐responsive genes. As molting progresses, decreasing m^6^A levels trigger the degradation of key molting regulators EcR and Cht10. eIF3‐S6 binds to m^6^A‐modified *EcR* and *Cht10* transcripts, forming LLPS condensates that protect these low‐m^6^A mRNAs from excessive degradation, thereby ensuring successful molting. Disruption of either condensate components or m^6^A modification abolishes phase separation, leading to molting defects (**Figure** **8**). This work reveals an essential coupling mechanism between m^6^A modification and LLPS for mRNA stabilization during insect molting.

RNA epigenetic modifications, particularly m^6^A, dynamically coordinate the expression of molting‐responsive genes in a timely manner during locust development. Disruption of the m^6^A pathway results in aberrant molting, ultimately leading to nymphal death. A recent study also demonstrated that the m^6^A pathway is essential for eclosion of *T. castaneum*.^[^ ^41^ ^]^ Knockdown of m^6^A writers prevents shedding of the pupal cuticle; however, the specific role of m^6^A in regulating the ecdysis process of pupal remains unclear. In *Drosophila*, ≈40% of embryos cannot reach the larval stage when maternal *Mettl3* and *Mettl14* were simultaneously removed.^[^ ^42^ ^]^ Similarly, m^6^A impacts caste differentiation and larval development of *Apis mellifera*.^[^ ^43^ ^]^ Mettl3 and Mettl14‐mediated m^6^A modification suppresses juvenile hormone degradation of *Plutella xylostella* to minimize fitness costs in response to a pathogenic attack.^[^ ^44^ ^]^ Collectively, m^6^A modifications are believed to play a crucial and evolutionarily conserved role in insect development.

Our results also highlight the key linkage between ecdysone signaling and the m^6^A machinery. Ecdysone signaling, a central regulator of molting (Figures S10A and S12A, Supporting Information), modulates the expression of RNA methylation enzymes. Injection of 20E can activate *Mettl3* but repress *Alkbh5* expression levels (Figure S11B,E, Supporting Information), although it has no effect on *Mettl14* and *Wtap* expression (Figure S11C,D, Supporting Information). Dual‐luciferase assays revealed that *Mettl3* promoter drove significantly higher expression than the reference reporter (Figure S12A, Supporting Information). However, 20E treatment did not significantly enhance the transcriptional activity of the *Mettl3* promoter in S2 cells (Figure S12B, Supporting Information). These results suggest that 20E regulates *Mettl3* expression independently of its promoter activity, but via alternative mechanisms. Furthermore, *Mettl3* knockdown suppressed the transcription of 20E nuclear receptors, early 20E‐response genes, and chitin metabolic genes in locust integuments, whereas SAM injection had the opposite effect, upregulating their expression (Figure S13B,C, Supporting Information). Thus, m^6^A modification may serve as an intermediate junction gate responsive to ecdysone signaling, coordinating the expression of molting effectors.

The m^6^A reader eIF3‐S6, as a molecular sensor, can induce the LLPS in response to low m^6^A modification levels. eIF3 is generally considered a translation initiation factors that stimulate both the recruitment of the initiator tRNA, Met‐tRNA_i_^Met^, and mRNA to the 40S ribosomal subunit and subsequent scanning of the mRNA for the AUG start codon.^[^ ^45^ ^]^ And there are barely studies on its dual functionality as both an m^6^A reader and LLPS regulator. Our mass spectrometry analysis identified eIF3‐S6 as a novel m^6^A reader that binds with molting‐responsive RNAs and exhibits phase separation capability. Like other characterized m^6^A readers, all three YTHDF proteins (YTHDF1, YTHDF2, YTHDF3) share a C‐terminal YTH domain and an N‐terminal IDR, enabling them to undergo LLPS.^[^ ^46^ ^]^ These findings reveal a novel m^6^A reader‐driven phase separation, underlying the alternative functions of eIF3.

Phase separation formation and m^6^A modification levels exhibit a pattern of mutual increase and decrease for mRNA stabilization. During molting, when m^6^A levels of *EcR* and *Cht10* are at their lowest, the m^6^A reader eIF3‐S6 undergoes phase separation, forming liquid droplet granules that recruit low‐abundance m^6^A‐modified mRNAs. When pre‐molting and post‐molting stages, elevated m^6^A modifications coincide with phase separation dissolution and serve as a critical regulatory mechanism for fine‐tuning the expression of molting‐responsive transcripts. Similarly, phase separation is involved in the process of RNA modifications, while RNA modifications reciprocally provide a regulatory mechanism for LLPS.^[^ ^47^, ^48^, ^49^, ^50^ ^]^ In vitro evidence suggests that high RNA levels keep RNA‐binding proteins soluble, whereas low RNA levels promote aberrant protein aggregation, leading to the formation of pathological gel‐like condensates in cells.^[^ ^51^ ^]^ Specifically, m^6^A‐modified mRNAs are actively recruited into various membraneless organelles through YTHDF proteins, enabling compartment‐specific regulation of mRNA stability and translation.^[^ ^52^ ^]^ These findings collectively demonstrate that the dynamic interplay between m^6^A modifications and LLPS serves as a reversible regulatory switch that precisely modulates transcriptional plasticity.

In conclusion, our findings propose a novel model in which m^6^A modified *EcR* and *Cht10* mRNAs instruct a phase‐switch of eIF3‐S6 granules, regulating mRNA stability during the molting process in locusts. This work underscores a functionally coupled regulation system and highlights the orchestrated dynamics of interconnected multi‐step processes governing the pre‐molt to molting transition.

## 4 Experimental Section

### Insects

The migratory locusts used in this study were reared at the College of Life Sciences, Capital Normal University, Beijing, China. Nymphs were reared in large boxes (40 cm × 40 cm × 40 cm) under a 14:10 light/dark photo regime at 30 °C ± 2 °C and provided with fresh wheat seedlings as food.

### RNA Extraction and Quantitative PCR

Total RNA was extracted from integuments using TRIzol reagent (Invitrogen). The concentration and purity of RNA were assessed with a Nanodrop ND‐1000 spectrophotometer to verify RNA integrity. cDNA was synthesized using the PrimeScriptTM RT Reagent Kit with gDNA Eraser (TaKaRa). mRNAs were subjected to qPCR by using the Real Master Mix Kit (Tiangen) on a LightCycler 480 instrument (Roche). The PCR data were analyzed by the 2^−ΔΔCt^ method, and β‐actin was used as the endogenous control. The primers for qPCR are listed in Table S1 (Supporting Information).

### RNA m^6^A Methylation Assay

The EpiQuik m^6^A RNA Methylation Quantification Kit (Epigentek) was used to assess the content of m^6^A in total RNA, as previously reported.^[^ ^53^ ^]^ Briefly, 200 ng of sample RNA, in conjunction with the m^6^A standard, were loaded into designated wells using RNA high‐binding solution. Subsequently, specific capture and detection antibodies were used to capture and assess m^6^A levels. The colorimetric m^6^A levels were quantified by measuring the absorbance of each well at an optical density of 450 nm, and the results were calculated based on the standard curve.

### LC‐MS/MS

LC‐MS/MS for determination of m^6^A/A ratio was performed as previously described.^[^ ^54^ ^]^ PolyA+ RNA was extracted from integuments of locusts at the early‐stage, mid‐stage, late‐stage of the 4^th^ instar and early‐stage of the 5^th^ instar using NEBNext Poly(A) mRNA Magnetic Isolation Module (NEB). Single nucleosides were generated through treatment with nuclease P1 (Sigma) and alkaline phosphatase (Sigma), and then quantified using LC‐MS/MS. The ratio of nucleoside‐to‐base ion mass transitions was calculated based on the measured concentrations.

### m^6^A Dot Blot

m^6^A dot blots were performed as previously described with slight modifications.^[^ ^55^ ^]^ Total RNAs (2500 ng) were double‐diluted and spotted onto a nylon membrane (Invitrogen). The membrane was then UV cross–linked, blocked and incubated with anti‐m^6^A antibody (Abcam, 1:1250) overnight at 4 °C. Subsequently, horseradish peroxidase‐conjugated goat anti‐rabbit IgG (Easybio, 1: 5000) was added to the blots for 2 h at room temperature and the membrane was detected by an ECL Western Blot Kit (Invitrogen). The same 2500 ng RNAs were spotted on the membrane, stained with methylene blue (Macklin) for 15 min, rinsed in PBST, and imaged.

### RNAi Assay

Double‐stranded RNAs (dsRNAs) of green fluorescent protein (*GFP*), *Mettl3*, *Mettl14*, *Wtap*, *Alkbh5*, *Ythdc1*, *Ythdf2*, *eIF3‐S6*, *EcR*, and *Cht10* were synthesized using T7 RiboMAX Express RNAi System (Promega) in accordance with the manufacturer's protocol. The injection and sampling methods of locusts were carried out as described previously.^[^ ^56^ ^]^ The 2‐day‐old fourth instar nymphs were injected with 5 µg of dsRNA into the second ventral segment of the abdomen. Control nymphs were injected with an equivalent amount of ds*GFP*–RNA. The effect of RNAi on relative mRNA expression levels 48 h after injection was investigated by qPCR. The nymphs that typically showed abnormal ecdysis were used for hematoxylin and eosin staining and chitin staining. The primers used for the synthesis of dsRNAs targeting *GFP*, *Mettl3*, *Mettl14*, *Wtap*, *Alkbh5*, *Ythdc1*, *Ythdf2*, *eIF3‐S6*, *EcR*, and *Cht10* are listed in Table S1 (Supporting Information). The nucleotide sequences used for RNAi are listed in Table S2 (Supporting Information).

### MeRIP‐seq

The methylated m^6^A RNA immunoprecipitation (MeRIP) was conducted following the protocol provided by using the m^6^A MeRIP kit protocol (Bersinbio). Briefly, total RNA was isolated and purified using the RNA Clean & Concentrator kit (Zymo). Then the RNA samples were incubated with 5 µL of anti‐m^6^A antibody (Abcam), or IgG (Bersinbio) that had been pre‐conjugated to protein G‐magnetic beads in 500 µL IP buffer at 4 °C for 2 h. The IP complex was treated with proteinase K at 55 °C for 30 min. Libraries were prepared using the NEBNext Ultra II Directional RNA Library Prep Kit for Illumina (NEB) for both IP and input RNA, and sequenced on the Illumina HiSeq point series platform. Each time point was performed in three biological replicates. Six integuments were involved in one biological replicate.

### Data Processing

The raw sequencing data were filtered, and the cleaned data were mapped to the locust genome utilizing STAR 2.7.10a software. The differential gene expression between samples was quantified at the gene level employing RSEM v1.3.1. The differential analysis of the count data was executed with the DESeq2 (R version for 4.2). The GO enrichment analysis was conducted by the GOseq R package, and gene length bias was corrected. The significance analysis was performed by Fisher's Exact Test.

### RNA Immunoprecipitation (RIP) Assays

The RIP assay was performed using a Magna RIP Quad Kit (Millipore) in accordance with the manufacturer's instructions. Ten integuments were collected and homogenized in ice‐cold RIP lysis buffer. The homogenates were stored at −80 °C overnight. A total of 10 µg of anti‐eIF3‐S6 antibody (Genscript Biotech Corporation) or normal rabbit IgG (Millipore), which served as a negative control, was pre‐incubated with magnetic beads. The frozen homogenates were thawed and centrifuged, and the supernatant was incubated with the magnetic bead–antibody complex at 4 °C overnight. Concurrently, a 30 µL portion of the sample lysate was reserved as “input”. The immunoprecipitated RNAs were reverse‐transcribed into cDNA using SuperScript IV (Invitrogen). qPCR was performed to quantify the expression levels of *EcR* and *Cht10*. To normalize the relative expression levels, the supernatants of the RIP lysate (input) and the IgG controls were assayed for specificity of RNA‐protein interactions.

### Protein Expression and Fluorescence Recovery after Photobleaching (FRAP)

The full‐length of eIF3‐S6 was cloned into the vector pET28a to generate the recombinant protein. The primers are presented in Table S1 (Supporting Information). The recombinant protein was expressed in Escherichia coli BL21 (DE3) within LB medium at 16 °C and purified using Ni‐NTA. Subsequently, the target protein was diluted to 10 µmol L^−1^ and dispensed into the 96‐well plate. 2 µL of Alexa Fluor 488 fluorescent dye (Invitrogen) was added to each protein sample. After thorough mixing, it was allowed to stand for 5 min to enable the protein to fully bind the dye. The culture plate was placed under a laser scanning confocal microscope, and the target area was irradiated with a laser beam to instantaneously photobleach the fluorescently labeled protein in the designated area. Thereafter, real‐time imaging and recording of the bleached area were conducted using a fluorescence microscope, and the real‐time recovery process of the protein in the target area after photobleaching was observed. A specific time interval was set to collect the fluorescence signal intensity and record the variations in fluorescence intensity. The fluorescence recovery rate was calculated by analyzing the variations in fluorescence signal intensity over time.

### NaASO_2_ and 1,6‐Hexanediol Treatment

To test the effect of NaASO_2_ and 1,6‐Hexanediol on the phase separation of eIF3‐S6, NaASO_2_ (Sigma) (10 mg mL^−1^) or 1,6‐hexanediol (Sigma) (1 mol L^−1^) was gently added into the recombinant eIF3‐S6 premixed with Alexa Fluor 488 fluorescent dye in 96‐well plates. The fluorescent signal of the target protein was observed under the confocal microscope. The size and frequency of droplet condensates were quantified during LLPS by R and ggforce (Figure S9, Supporting Information).

### B‐isox Precipitation and Mass Spectrometry

These assays were performed as previously described with slight modifications.^[^ ^57^ ^]^ Briefly, eight integuments were homogenized in ice‐cold RIPA lysis buffer and incubated on ice for 10 min. The supernatant was then collected after centrifugation at 14,000 rpm for 10 min. 10% of the supernatant was reserved as input. Subsequently, the remaining lysate was supplemented with b‐isox (Sigma), incubated on ice for 20 min, and centrifuged at 14,000 rpm for 10 min at 4 °C to separate the supernatant and precipitate. The RNA and protein from both the supernatant and precipitate were extracted using Trizol as above.

For mass spectrometry, samples were run on a 10% SDS‐PAGE gel and subjected to Coomassie staining. Total bands were then cut for each sample and submitted to the BGI Genomics for analysis.

### Lifespan Assay

After the injection of dsRNA for *Mettl3*, *Mettl14*, *Wtap*, *Alkbh5*, *Ythdc1*, and *eIF3‐S6*, the locusts were raised in cages and fed wheat seedlings. The number of dead insects was recorded daily, and the survival rate was calculated within eight days in one group. The control group was injected with *GFP* dsRNA.

The lifespan assay for the injection of 1,6‐hexanediol or SAM on locusts was performed as above. The 2‐day‐old fourth instar nymphs were injected with 1,6‐hexanediol (2 µL,1 mol L^−1^) or SAM (2 µL, 80 µmol L^−1^) in the second ventral segment of the abdomen and reared in cages for eight days, dead locusts were scored and counted every day. The control group was injected with PBS.

### Immunohistochemistry

The colocalization of eIF3‐S6 and G3BP1 was detected in the integument using immunohistochemistry. The integument was fixed in 4% paraformaldehyde overnight. Paraffin‐embedded integument tissue slides (5 mm thick) were deparaffinized in xylene and rehydrated with an ethanol gradient. The samples were blocked with 5% (wt/vol) skimmed milk for 30 min at room temperature and then incubated with affinity‐purified rabbit antibodies against eIF3‐S6 (1: 500, Genscript Biotech Corporation) and G3BP1 (1: 1000, Abclone). An Alexa Fluor 488‐conjugated goat anti‐rabbit antibody (1: 500, Life Technologies) was used as the secondary antibody for eIF3‐S6 and G3BP1 staining. Fluorescence was detected using an LSM 780 confocal laser‐scanning microscope (Zeiss). Images for both positive staining and negative controls were captured under the same conditions. The specificity of the antibody against eIF3‐S6 or G3BP1 was evaluated (Figure S10, Supporting Information).

### Co‐Immunoprecipitation (CO‐IP) and Western Blot Analysis

For the interaction assay of eIF3‐S6 and G3BP1 in locusts, CO‐IP was conducted using a Dynabeads Protein G Immunoprecipitation Kit (Invitrogen) in accordance with the manufacturer's guidelines. Initially, 5 µg of either rabbit anti‐eIF3‐S6 or rabbit anti‐G3BP1 antibody was conjugated to 50 µL Dynabeads Protein G (Novex) for 30 min at room temperature. Subsequently, 400 µL of total protein extracted from the integuments of locusts using RIPA lysis buffer was added to the mixture. ≈10% of the total protein was stored as input. Rabbit IgG (ABclone) served as a negative control. Following three times of washing with washing buffer, the antibody‐protein complex was dissociated from the beads using elution buffer for subsequent Western blot analysis with either rabbit anti‐G3BP1 or rabbit anti‐eIF3‐S6.

Total proteins for Western blot analysis were first extracted by using TRIzol reagent. The proteins were subjected to gel electrophoresis and then transferred to polyvinylidene difluoride (PVDF) membranes (Millipore). The membranes were blocked with 5% skimmed milk at room temperature for 2 h, followed by incubation with primary antibody (rabbit anti‐eIF3‐S6, 1: 500; rabbit anti‐G3BP1, 1:2000) in PBST overnight at 4 °C. Goat anti‐rabbit IgG was used as secondary antibody (CWBIO, 1:5000). Protein bands were detected by SuperSignal West Femto (Thermo Fisher Scientific). The intensities of the Western blot signals were quantified using Image J.

### mRNA Stability Assay

The integument tissues of the locust nymphs were immediately dissected and snipped in a culture dish supplemented with Schneider's *Drosophila* Medium (Gibco) to release the epidermal cells. The cell suspension was transferred to a cell culture bottle and cultured for 3 to 6 h at 30 °C. Subsequently, the integument cells were transferred to 6‐well plates and treated with 0.2 mm actinomycin D (G‐CLONE) for 0, 2, 4, 6, and 8 h. The samples were then collected for total RNA extraction and cDNA synthesis, which were performed according to the methods described above. qPCR was conducted to quantify mRNA levels.

### GLORI‐seq

RNA protection, deamination, and deprotection were performed according to a method that was described previously.^[^ ^58^ ^]^ Briefly, RNA fragments were generated through incubation at 95 °C for 10 min in fragmentation buffer (Invitrogen). 100 ng of fragmented RNA were incubated in a 20 µL reaction system containing 2.5 µL glyoxal (Sigma) and 2.5 µL nitrite (Sigma) to induce selective deamination of unmethylated adenosine. RNA was purified by ethanol precipitation and RNA Clean & Concentrator kit (Zymo). Deamination efficiency was quantitatively assessed by LC‐MS/MS. Sequencing libraries were constructed from immunoprecipitated (IP) and input RNA samples using the NEBNext Ultra II Directional RNA Library Prep Kit (NEB), and sequenced on Illumina Hiseq X‐ten with paired‐end (PE) run and read lengths of 2  ×  150 base pairs.

### In Vitro Mutagenesis Assay

The full‐length coding sequence (CDS) of *EcR* and a ∼1.5 kb fragment flanking the target modification site in *Cht10* were separately cloned into pAC5.1/ V5‐His b (pAc5.1b) vector using the EcoR V and Not I sites. Site mutations were gained by using a KOD‐Plus‐Mutagenesis Kit (TOYOBO) following the manufacturer's instructions. The primers used for the construction of recombinant plasmids are listed in Table S4 (Supporting Information).

S2 cells were cultured at 28 °C in a 24‐well plate for 24 h before transfection. The constructed plasmids were cotransfected with pAC5.1‐*Mettl3* and pAC5.1‐*eIF3‐S6* into the S2 cells by Lipofectamine 3000 reagent (Invitrogen). Cells were harvested 48 h post‐transfection and lysed in RIP buffer supplemented with RNase inhibitor. RNA‐protein complexes were immunoprecipitated using anti‐m⁶A (Abcam) or anti‐eIF3‐S6 (Genscript Biotech Corporation) antibodies. Co‐precipitated RNA was quantified by qPCR with normalization to β‐actin as an endogenous control.

### Luciferase Reporter Assay

We amplified a ≈3.0‐kb genomic region upstream of the *Mettl3* translation start site from the locust genomic DNA as the candidate promoter. This region was cloned into the pGL4.10 luciferase reporter vector (Promega). For normalization, the pGL4.73 vector, which contains the *Renilla* luciferase (*hRluc*) gene driven by the SV40 enhancer/promoter, was used as an internal control. S2 cells were co‐transfected with pGL4.10‐*Mettl3* promoter construct, pGL4.73 reference plasmid, and Pac5.1b*‐EcR* plasmid after treatment with 20E or PBS (control) using Lipofectamine 3000 (Invitrogen). Luciferase activity was measured 48 h post‐transfection using the Dual‐Luciferase Reporter Assay System (Promega) on a GloMax 96 Microplate Luminometer. Data were presented as the ratio of firefly luciferase to *Renilla* luciferase activity.

### Statistical Analysis

All data were analyzed using IBM SPSS statistics 19. The differences between treatments were compared using either Student's *t*‐test or one‐way analysis of variance (ANOVA) followed by Tukey's test for multiple comparisons. The differences of the survival curves were calculated using the Kaplan–Meier statistical analysis. All results were expressed as means ± SEM.

## Conflict of Interest

The authors declare no conflict of interest.

## Supporting information



Supporting Information

## Data Availability

The data that support the findings of this study are available from the corresponding author upon reasonable request.;
