# Supplementary material for: Dynamic eIF3‐S6 Phase Separation Switch Instructed by m6A Modification Drives the Molting of Locusts
Source: Adv Sci (Weinh). 2025 Jul 28;12(39):e10505. doi: 10.1002/advs.202510505 (PMC12533359; doi:10.1002/advs.202510505)
Supplement: Supplementary file 1 — Supporting Information [file ADVS-12-e10505-s001.docx]

**Figure S1.** **Comparative sequence and structural analyses of RNA demethylases between locusts and other species.** A) Phylogenetic analysis of RNA demethylases among five locust species, Drosophila, mice, and humans orthologs. B) Comparative analysis of the conserved structural and functional domains in RNA demethylases between locusts and Drosophila / mice / humans. C) AlphaFold-predicted three-dimensional structures of RNA demethylases in locusts and Drosophila.

**Figure S2. m^6^A RNA modification might be associated with the molting process in the locust.** A) The mRNA m^6^A levels by HPLC-MS assays in the integuments at the early and late-stages of the 3^rd^ instar nymph, as well as at the early-stage of the 4^th^ instar nymph. B) The mRNA levels of *Mettl3*, *Mettl14*, *Wtap*, *Alkbh5* after the injection of their dsRNA into the locust integuments by qPCR, respectively. C, D) The total RNA m^6^A levels in the integuments at the late-stage of the 4^th^ instar nymphs after injection of ds*Mettl3* (C) or ds*Mettl14* (D) by dot blot assay. The dot blot bands were quantified through densitometry. Different letters indicate statistically significant differences between groups using one-way ANOVA (A, Tukey’s multiple comparisons test, *p* < 0.05). *P* values were determined by a two-tailed unpaired *t*-test (B, C and D). Data are presented as mean ± SEM. * *p* < 0.05, ****p* < 0.001.

**Figure S3.** **MeRIP-seq and RNA-seq uncovers the differential expressed m^6^A-genes associated with molting.** A) Principal component analysis (PCA) on the MeRIP-seq of the integuments at the early-stage, mid-stage, late-stage of the 4^th^ instar nymph and early-stage of the 5^th^ instar nymph. B) PCA analysis on the RNA-seq of the integuments in ds*Mettl3* and ds*GFP* group. C) Heat-map indicating genes with significant reductions in both m^6^A enrichment (late vs. early stage, MeRIP-seq) and transcript levels (ds*Mettl3*- vs. ds*GFP*-injected nymphs, RNA-seq). These genes were categorized into 6 clusters by hierarchical clustering using a complete linkage algorithm. Genes from Cluster A and cluster C indicate the top candidate targets (*EcR* and *Cht10*) and several m^6^A regulatory components, including two readers (*Hnrnp* and *eIF3-S6*), an eraser (*Alkbh5*), and rRNA methyltransferases (*Mettl5* and *Zcchc4*). D) The motif identified from the m^6^A peaks in the m^6^A IP group.

**Figure S4.** Relative expression levels of the other 5 reader genes (*Ythdc1* and *Ythdf2* as Yth homologs; *Hnrnp* and *Hnrnpc* as Hnrnp homologs; *Igf2bp3* as an Igf2bp homolog) searched from the locust genome in the integument of early-stage, mid-stage, and late-stage of the 4^th^ instar nymph and early-stage of the 5^th^ instar nymph by qPCR (n = 6). Different letters indicate statistically significant differences between groups using one-way ANOVA (Tukey’s multiple comparisons test, *p* < 0.05). The qPCR data are shown as mean ± SEM.

**Figure S5.** The mRNA levels of *EcR* and *Cht10* (B) as well as *eIF3-S6* (C) after the injection of their dsRNA into the locust integuments, respectively. *P* values were determined by a two-tailed unpaired *t*-test. Data are presented as mean ± SEM. *** *p* < 0.001.

**Figure S6.** The strategy used to generate the plasmid expression vectors *EcR* WT / *EcR* mutants and *Cht10* WT / *Cht10* mutants.

**Figure S7.** **The sequence properties of m^6^A-binding protein eIF3-S6.** A) This protein comprises an eIF domain, a low-complexity domain and a PINT domain by computational predictions. B) The purified recombinant eIF3-S6 protein by western blot. Size markers are labeled left of the gel.

**Figure S8. Neither Ythdc1 nor Ythdf2 played a detectable role in the locust molting.** A, C) The mRNA levels of *Ythdc1* and *Ythdf2* after the injection of their dsRNA into the locust integuments, respectively. B, D) The effects of silencing *Ythdc1* (B) or *Ythdf2* (D) on the mortality and molting of locusts, with the green arrow indicating the critical time for molting. *P* values in qPCR were determined by a two-tailed unpaired *t*-test. *P* values in survival curve were determined by the Kaplan-Meier statistical analysis. Data are presented as mean ± SEM. ** *p* < 0.01; *** *p* < 0.001.

**Figure S9.** The size and frequency of droplet condensates were quantified during LLPS by R platform and ggforce.

**Figure S10.** Validation of the polyclonal antibodies against the eIF3-S6 and G3BP1 proteins. A, B) RNAi-induced knockdown of eIF3-S6 (A) and G3BP1 (B) was used to validate the antibody specificity. RNAi *GFP* was used a control.

**Figure S11.** **Injection of 20-hydroxyecdysone (20E) affects the molting development and m^6^A modification of locusts.** A) 20E initiates the molting of locusts in advance. B) The mRNA levels of *Mettl3*, *Mettl14*, *Wtap*, *Alkbh5* were detected after the injection of 20E by qPCR, respectively. *P* values in qPCR were determined by a two-tailed unpaired *t*-test. *P* values in survival curve were determined by the Kaplan-Meier statistical analysis. Data are presented as mean ± SEM. * *p* < 0.05, ** *p* < 0.01.

**Figure S12. 20E regulates** ***Mettl3* expression independently of its promoter activity.** A) Transcriptional activity of the *Mettl3* promoter assessed by dual luciferase activity reporter assay. S2 cells were co-transfected with pGL4.10 reporter plasmids containing the *Mettl3* promoter region and a reference reporter plasmid pGL4.73. The empty pGL4.10 plasmid was used as a negative control. *p* < 0.001, n = 6. B) Effect of 20E on *Mettl3* promoter activity in *EcR*-expressing S2 cells. S2 cells were co-transfected with pGL4.10-*Mettl3* promoter construct, pGL4.73 reference plasmid, and Pac5.1b*-EcR* plasmid after treatment with 20E or PBS (control). n = 6, *p* values were determined by a two-tailed unpaired *t*-test. Data are presented as mean ± SEM. ns, not significant.

**Figure S13. The effects of m^6^A modification on 20E signaling pathway.** A) 20E signaling pathway controlling the molting of insect. B, C) The mRNA levels of 20E nuclear receptors, early 20E-response genes and chitin metabolic genes in 20E signaling pathway were detected after the injection of ds*Mettl3* or SAM by qPCR (n = 6), respectively. *P* values in qPCR were determined by a two-tailed unpaired *t*-test. Data are presented as mean ± SEM. * *p* < 0.05, ** *p* < 0.01, *** *p* < 0.001, ns, not significant.

**Table S1. Primers used in this study.**

**Table S2. The sequences of gene RNAi fragment used in this study.**

**Table S3. The motif identified from the m^6^A peaks in the m^6^A Input and IP group.**

**Supplementary Tables**

**Table S1. Primers used in this study.**

| **Primer name** | **Sequence (5’-3’)** |
| --- | --- |
| Primers used for qPCR | |
| qMettl3-F | TCCCTACCGAAGACAAAG |
| qMettl3-R | AAGCAACGAAGCAGGAGT |
| qMettl14-F | TGAAGTAGCAGCAGCACG |
| qMettl14-R | TGGAGTGGGATGGATTGT |
| qWtap-F | ACAACAGGAACTGCGTAA |
| qWtap-R | CTCACTTGTCCGTAAACC |
| qAlkbh5-F | TAACAACCCACGGAAACG |
| qAlkbh5-R | GGCTCTGACCCAGGACTT |
| qN2un2-F | ATGAACGACAACCCTAAA |
| qN2un2-R | AGACATCTGCTCCCAAAA |
| qTet2-F | CTGTGCCCATTCTCATCG |
| qTet2-R | CTGGCTCGTCCTCTTTGC |
| qTrmt6-F | TGAGTGGAAGTGGAGGTT |
| qTrmt6-R | CTCTTTCGGAGATTGCTG |
| qTrmt61-F | ACGGCGGCAGGTTTGTAT |
| qTrmt61-R | TCAGTCTTGGCGAGGTGG |
| qMettl1-F | TGGCTGTGGTTATGGTGG |
| qMettl1-R | TCATACTTGCCAGGGTGC |
| qWdr4-F | AAGGAGCCATTAGCAGTA |
| qWdr4-R | ATAGAAGCAAGGCACAAC |
| qEcR-F | TCCGAAGAAGATTTAAGACG |
| qEcR-R | CTTCCCGTAGCAGTTTGT |
| qCht10-F | CGTGGTTATGTGCGAGAC |
| qCht10-R | TCAACACGCTGTGAAGAG |
| qeIF3-S6-F | AGCCTCAAGGAGTTAGTG |
| qeIF3- S6-R | GTAAGGCACGCTGTCAAG |
| qYthdc1-F | GCTCCTCAAGACCTAATC |
| qYthdc1-R | ACTTTGGACGCACTGATT |
| qYthdf2-F | TTCTCGCAAACCACATTA |
| qYthdf2-R | ATTCCCATATCTACACTACCA |
| qHnrnp-F | ATTTCTCGCCAGGATGTT |
| qHnrnp-R | ACTGGGTCGTAGTCGTCA |
| q-Hnrnpc-F | TCAGGACCCGCAGGCCGTCA |
| q-Hnrnpc-R | AACTTGCCCCAGCACCGT |
| qIgf2bp3-F | CCCGAGATTAGACAGGCAGTA |
| qIgf2bp3-R | GATGGTTCCCTTCACAGTTATT |
| qRxr-F | CCTCAGAGCAGGTTGGAATGA |
| qRxr-R | CGCAAGCAGCCAAGTTCAGT |
| qHr3-F | GAAGGTGGAGGACGAGGTG |
| qHr3-R | GCCGTTGTAGGCGGACTG |
| qHr4-F | GGGCCGTACAATAACAATGG |
| qHr4-R | GCCACCTCGACCTTGATTTA |
| qE74-F | ATTATTGCCCACGCTACATTA |
| qE74-R | GGTAGACGAGTCGCTGG |
| qBr-c-F | GCGGGAGAGGCGAATGT |
| qBr-c-R | GGTGCGTGCTCGCTTGTT |
| qTres2-F | TACGGGACCGTAAGGTGTTGG |
| qTres2-R | CCACATTCTGCATTTTTGCTTATAC |
| qGna-F | TACGGGACCGTAAGGTGTTGG |
| qGna-R | CCACATTCTGCATTTTTGCTTATAC |
| qPgam-F | TACGGGACCGTAAGGTGTTGG |
| qPgam-R | CCACATTCTGCATTTTTGCTTATAC |
| qUap-F | TACGGGACCGTAAGGTGTTGG |
| qUap-R | CCACATTCTGCATTTTTGCTTATAC |
| qChs1-F | TACGGGACCGTAAGGTGTTGG |
| qChs1-R | CCACATTCTGCATTTTTGCTTATAC |
| qNag-F | GGTGGGAGATAATGCTACA |
| qNag-R | AAAGACATCAGAGTCGAAAA |
| qβ-actin-F | CGAAACCTTTAATACCCCAG |
| qβ-actin-R | CCATCACCAGAATCCAACAC |
| Primers used for RNA interference | |
| T7F-dsMettl3 | TAATACGACTCACTATAGGACTCCTGCTTCGTTGCTT |
| T7R-dsMettl3 | TAATACGACTCACTATAGGCTTTGCTGTAGGCTTGCT |
| F-dsMettl3 | ACTCCTGCTTCGTTGCTT |
| R-dsMettl3 | CTTTGCTGTAGGCTTGCT |
| T7F-mettl14 | TAATACGACTCACTATAGGAAGTAGCAGCAGCACG |
| T7R-mettl14 | TAATACGACTCACTATAGGGAGCGAAGAGTCACCA |
| F-mettl14 | AAGTAGCAGCAGCACG |
| R-mettl14 | GAGCGAAGAGTCACCA |
| T7F-dsWtap | TAATACGACTCACTATAGGTTGAG GGCGATTTAGC |
| T7R-dsWtap | TAATACGACTCACTATAGGGGTGG GTGGGAGACAT |
| F-dsWtap | TTGAGGGCGATTTAGC |
| R-dsWtap | GGTGGGTGGGAGACAT |
| T7F-Alkbh5 | TAATACGACTCACTATAGGGATTGGGTTCACAGTAT |
| T7R-Alkbh5 | TAATACGACTCACTATAGGTCTTGTTGCCTCGTAG |
| F-Alkbh5 | GATTGGGTTCACAGTAT |
| R-Alkbh5 | TCTTGTTGCCTCGTAG |
| T7F-dsEcR | TAATACGACTCACTATAGGCCAAGGTGGAACCAGAAA |
| T7R-dsEcR | TAATACGACTCACTATAGGTGCAGTCAGAAGGGCATA |
| F-EcR | CCAAGGTGGAACCAGAAA |
| R-EcR | TGCAGTCAGAAGGGCATA |
| T7F-dsCht10 | TAATACGACTCACTATAGGAATGATTTGGGCTTTAGACCTGG |
| T7R-dsCht10 | TAATACGACTCACTATAGGTGGGCATAGGATGGATTACAGAG |
| F-Cht10 | AATGATTTGGGCTTTAGACCTGG |
| R-Cht10 | TGGGCATAGGATGGATTACAGAG |
| T7F-dseIF3-S6 | TAATACGACTCACTATAGGATGGCGAAGTTTGATTTG |
| T7R-dseIF3-S6 | TAATACGACTCACTATAGGCTGGAAGACACTGCGTAA |
| F-dseIF3-S6 | ATGGCGAAGTTTGATTTG |
| R-dseIF3-S6 | CTGGAAGACACTGCGTAA |
| T7F-dsYthdc1 | TAATACGACTCACTATAGGACCAAGGCACAGACTAAA |
| T7R-dsYthdc1 | TAATACGACTCACTATAGGTCATTCCACGGATTGTAG |
| F-dsYthdc1 | ACCAAGGCACAGACTAAA |
| R-dsYthdc1 | TCATTCCACGGATTGTAG |
| T7F-dsYthdf2 | TAATACGACTCACTATAGGCATCGGACCCATACAACA |
| T7R-dsYthdf2 | TAATACGACTCACTATAGGTTATCAGACGGGCTTTGC |
| F-dsYthdf2 | CATCGGACCCATACAACA |
| R-dsYthdf2 | TTATCAGACGGGCTTTGC |
| Primers used for eIF3-S6 recombinant expression | |
| F-eIF3-S6 | ATGGCGAAGTTTGATTTGAC |
| R-eIF3-S6 | TCAAAACTCTGGAGCTCCCC |
| eIF3-S6-EcoRⅠ-F | ATGGGTCGCGGATCCGAATTCATGGCGAAGTTTGATTTGAC |
| eIF3-S6-NotⅠ-R | TGGTGGTGCTCGAGTGCGGCCGCTCAAAACTCTGGAGCTCCCC |
| Primers used for EcR and Cht10 sites mutation | |
| EcR-EcoR V-F | GTGGTGGAATTCTGCAGATATCATGGAGCGCGGCATGAGCGT |
| EcR-Not I-R | CCCTCTAGACTCGAGCGGCCGCTTATGGGATCACATCCCA |
| EcR-A335-F | AGTCTGCACGGTTACAGCGCGGACAGCTGTG |
| EcR-A335-R | GCTTAGTGGCGAGAGGTCCTCCCGGCCTGA |
| EcR-A1354-F | TCTGGACCAATTTTTGCAAAGTTGTTGTCA |
| EcR-A1354-R | CTTAGGACGCCGCCTGTTGTCCACATATGC |
| EcR-A1389-F | CTTACTGACCTGCGTACTCTAGGAAACCAG |
| EcR-A1389-R | AACTGACAACAACTTTGCAAAAATTGTTCC |
| EcR-A1412-F | AACCAGACCTCAGAAATGTGCTTCTCTCTCAAA |
| EcR-A1412-R | TCCTAGAGTACGCAGTTCAGTAAGAACTGA |
| Cht10-EcoR V-F1 | TGGTGGAATTCTGCAGATATCATGTGGGCACTTGATTTAGATGATTTT |
| CHT10-Not I-R1 | GCCCTCTAGACTCGAGGCGGCCGCGCCAACCAGCAGTTTAT |
| Cht10-EcoR V-F2 | TGGTGGAATTCTGCAGATATCATGAAGGCTGTAGTTGATGCC |
| CHT10-Not I-R2 | GCCCTCTAGACTCGAGGCGGCCGCAACTTTGCTCGGTGATAC |
| Cht10-A1923-F | GAAGAAGGCCTTATGGAAGATCCAGAAGACTGTAGG |
| Cht10-A1923-R | ACTACAGGCTACCTCTGACTGATTCTCTGGTTCAAC |
| Cht10-A7354-F | TCTGAAGAGCCTGATAATCGCATTCATCCCACAACTACT |
| Cht10-A7354-R | TCCTGGTTTCTCAGTGGCCTCAATATCACAGTT |
| Cht10-A7545-F | CCACAGGGCCTTTACTGGAATAAGGATCATTGT |
| Cht10-A7545-R | ACAACTCTGTTCCATGAAGTCACCATATTGACA |
| Cht10-A7589-F | AATACAGCCTGCAGCAAGGAAGACAACTCTGTA |
| Cht10-A7589-R | TGAAGGCCAGTCACAATGATCCTTATTCCAGTA |

Red font indicates T7 promoter sequence.

**Table S2. The sequences of gene RNAi fragment used in this study.**

| **Gene name** | **Sequence (5’-3’)** |
| --- | --- |
| *Mettl3* | ACTCCTGCTTCGTTGCTTATGTGAAGTGTCGCTGACTCTGCCTACAAGCTCAAGCGAGCTCGCTGCTGCTGTCGGTAAACAGCTTAATAAAAGTGTTCCTCATCTTGCTGTGACAAATCTTTTGCAGAAATTTGCAACACAGCAGTTGATAAGTGTGAAAGAGAACAGCAAAGATGGAAAACCAGCACTAGATGTTGTTTCTGCAGAACACACCAAGCTTGTGGCCATGGTAAATGAGCTAGAAGGAGAAGAAAAAGCACGAACCTTAGAACAACCAACAGAGGAAGTTAATCGAAAAAGAAAATGTGAGGGGGAACTTGAGGGAGAACCTGCACCCAAGGCTATAAAGACTGCAGCCTCTGACAGAGATAAGGATCCAAAGGCTGCTGATATTATGTCACTTCTCTCCATGCCTTCAATACGTGAAAAGGAGAATAAGAAAGTTGGAGAAGAAATTTTAGATCTCCTCAGCAAGCCTACAGCAAAG |
| *Mettl14* | AAGTAGCAGCAGCACGCAGTTTTGTTTTTCTTTGGTGTGGATCCTCAGAGGGTTTGGACATGGGCAGAGTTTGCTTACGAAAATGGGGATTTCGTCGGTGTGAGGACATTTGTTGGATTCGTACAAATATTAACAATCCATCCCACTCCAAAAATTTAGAACCAAAGGCTGTTTTCCAGAGGACAAAGGAACATTGTCTGATGGGTATTAAAGGAACTGTCCGGCGCTCAACAGATGGAGATTTTATTCATGCAAATGTGGATATTGATCTAATTATCTCCGAGGAACCGGAGTATGGATCCTTGGAAAAACCTGTTGAAATATTCCATATTATTGAGCACTTCTGTTTGGGAAGTAGAAGAGTTGGTGACTCTTCGCTC |
| *Wtap* | TTGAGGGCGATTTAGCTCTGCAAAAAAGCTTTAGTGAAGAAGTGAAAAAATCCCAGTCTGAACTAGATGAATTTCTTCAAGATTTGGATGAGGATGTGGAAGGAATGCAGAGTACAATATATTATTTACAACAGGAACTGCGTAAAGCAAAGGAATCAAATGCTGCTCTACAACAAGAAAATGCTGCTTTAAAATCGGGTGGAGTGACAGCAACCGAGCACAATATCGGTTTACGGACAAGTGAGGTGGGAAGGGAATCGAGGCCACGGACGCCTGTGTGTCACAATGGGATAAGGGAGAAAGGGGAGGAAGATTCCAGAGGTTGGGATGATCGGACTCAAGGGTCAGATAGGCCAGTAACGAGAGAGACATCCCAGTCACCAGTCACTGAAAATGCAGCAGACCGTCCGACACGCCGAGATGTCTCCCACCCACC |
| *Alkbh5* | GATTGGGTTCACAGTATGGTCATTGAACCTCTCGTAAAAGCAGGAATTGTACCTGATGGCTTCATCAACTCTGCAGTCATTAATGATTATCAACCTGGAGGCTGCATAGTTTCTCATATTGATCCTATACACATATTTGATAGGCCAATTGTATCTGTCTCATTCATGAGTGACAGTGCTCTTAGCTTTGGCTGCAAGTTCAGTTTCAAGCCAATCAGAGTATCAAAGCCCATCTTATGCTTACCTTTGTCGCGAGGGTGCGTCACCATTCTCAGTGGGTTCGCAGCAGATGAAATTACGCATTGTGTGAGACCTGAAGATACCATTGCTAGGCGTGCTGTTATCTTACTGAGAAGGGTATTTCCAGATGCACCACGATTATCACCAAATGAAGTGATACCACCTGTGCCAATGAATGCATCTACGAGGCAACAAGA |
| *EcR* | CCAAGGTGGAACCAGAAAGACCTTTATCAAATGGGATAAAACCTGTAAGTCCTGAACAGGAAGAGCTTATACATAGGCTTGTGTACTTCCAGAATGAATATGAGTCTCCTTCCGAAGAAGATTTAAGACGAGTTACGAGTCAACCTACGGAAGGAGAGGACCAAAGTGATGTAAGGTTTCGACACATCACTGAGATCACAATATTAACTGTTCAACTAATTGTTGAATTTGCCAAGCGGTTGCCAGGATTTGACAAACTGCTACGGGAAGATCAGATAGCATTACTGAAGGCATGTTCCAGTGAAGTAATGATGTTCCGCATGGCACGACGCTATGATGTAAATTCAGACTCCATACTTTTTGCCAATAATCAGCCTTACACTAAGGATTCCTACAACCTTGCTGGTATGGGAGAAACGATAGAAGACATGTTGCGGTTCTGCAGACAGATGTATGCAATGAAGGTTGATAATGCAGAATATGCCCTTCTGACTGCA |
| *Cht10* | AATGATTTGGGCTTTAGACCTGGATGATTTCCGTAATGTGTGTTCTTGTGAGAAATATCCTCTACTGAAAACAATTAACAGAGTCCTAAGAGGATACCCAGGCCCTGGTCCTAACTGTGATATTGAGGCCACTGAGAAACCAGGATCTGAAGAGACTGATAATCGCATTCATCCCACAACTACTAAACCAACAACTAACAACTGGAATGTAATAAGTGGTGGCAGTGGTCTTGTTCCAAAAGATCCTACATGTGGGAACCGTCTGTTTGCTCCACATGATACAGACTGTAACAAATATTATCTCTGTCAATATGGTGACTTCATGGAACAGAGTTGTCCACAGGGACTTTACTGGAATAAGGATCATTGTGACTGGCCTTCAAATACAGACTGCAGCAAGGAAGACAACTCTGTAATCCATCCTATGCCCA |
| *Ythdc1* | CAAGGCACAGACTAAACAGAAGTCGCTCAAGGGAGAGAAAAAGCCCTCCACCGGTGCTAAAGCGTTCGAAATCGAGAGAGAAGTCAAAGTCTTATGATTACATAACGAAACTTAATTATTTATTCAGAGATGCAAGATTTTTTCTTATTAAGAGCAATAATGCAGAAAATGTAACCCTGTCAAAAGCCAAGGGTGTATGGTCCACGCTTCCACAGAATGAATCGAAGTTGAACCATGCTTACAGAGAATGTAGAAATGTCATTCTCATATTTTCTGTTAAAGAAAGTGGAAAATTTGCTGGCTTTGCACGATTAAGTGGAGAGTCAAGACGGGATGTATCACCAATTTCTTGGGTGCTGCCTCCAGGACTTTCAGCAAGGGCCTTAGGAGGTGTTTTCAGAGTGGATTGGGTTTGCCGCAAAGAATTGCCTTTCAGTGCAACTGTACATCTCTACAATCCGTGGAATGA |
| *Ythdf2* | CATCGGACCCATACAACATGTCAAATTACTATGGAACATCGTTTCCATACCAAGCATTTGGAGTGGGGGATGGTACTTGGTCGAATGGAGGTGATCCCATGACTTTTTTGGGTGGCTACGGTGGACAGATGGGCCATGATTCGTACAGTATGGACGGTATGTTTGGTGGTGGCGGCGGTGGGTTTGGAACAGCTTTCGGGCAGCCTGCTGCAGCAGCAGCAGCTGCCGCTTCCGGGTTCAACTACTTTCATGGCAATGGAGACTTTTCAACGTGGGGCAGCTCTGCTGCACCCATTTCTCGCAAACCACATTATGATGATTATTATAGGGGTGGAGATGGCATGTACACAGCACCTGGAGTTGGTGGTTCTGATGTAAAAGCTGTAGAGCAGGGAATGCAAGGTCTAGGACTTTCAGATACTAGTAGTGGCACTGGGAAAGTTTCTGATGGCAAGGACTTAAAACAGCAAAGCCCGTCTGATAAAAGTGGTAGTGTAGATATGGGAA |
| *eIF3-S6* | ATGGCGAAGTTTGATTTGACATCGACGTTAGGACAGTATTTGGACAGACATTTAGTTTTTCCGTTATTAGAATTTGTATCAGCGAAGCAGATATACGATGAAACGGAACTTTTGCAAGGGAAGCTGGATATTCTCAGCAAGACAAATATGGTTGATTATGCGATAGACATACGAAAGCAGTTATATCCGGATCAAGATGTGCCAGAGTCACTGAAACAGAAGAGAGCAGAAGTTGTAACACAGCTAGCAGTAATTCAGAAAGATGTGGCAACTGTACTCGAGATTATATCAAATGATGATGTAATGAACAAGATGGAGAATCTTAGAGATCCTGAGGCATTTGTGCAGAACCTTATAAAAGAACATCATTTCAATTTGAATATGATGGACAGTATGTACAAGTTAGCAAAGTATAGGTATGAATGTGGTAATTACGCAGTGTCTTCCAG |

**Table S3. The motif identified from the m^6^A peaks in the m6A Input and IP group.**

| **Motif** | **Input** | **IP** | **Foldchange** | ***P value*** |
| --- | --- | --- | --- | --- |
| Repeat | GG(A)AC | GG(A)AC | 117.0413 | 7.07e-09 |
| Repeat1 | G(C)GAC | A(C)GAC | 106.9987 | 1.82e-08 |
| Repeat2 | GG(A)AC | GG(U)AC | 106.0218 | 8.55e-08 |
| Repeat3 | G(C)GAU | G(G)GAC(U) | 100.9271 | 4.11e-08 |
